# Avian lungs: A novel scaffold for lung bioengineering

**DOI:** 10.1371/journal.pone.0198956

**Published:** 2018-06-27

**Authors:** Sean M. Wrenn, Ethan D. Griswold, Franziska E. Uhl, Juan J. Uriarte, Heon E. Park, Amy L. Coffey, Jacob S. Dearborn, Bethany A. Ahlers, Bin Deng, Ying-Wai Lam, Dryver R. Huston, Patrick C. Lee, Darcy E. Wagner, Daniel J. Weiss

**Affiliations:** 1 Department of Surgery, University of Vermont, Burlington, VT, United States of America; 2 Department of Medicine, University of Vermont, Burlington, VT, United States of America; 3 Rochester Institute of Technology, Rochester, NY, United States of America; 4 Department of Mechanical Engineering, University of Vermont, Burlington, VT, United States of America; 5 Department of Biology, University of Vermont, Burlington, VT, United States of America; 6 Comprehensive Pneumology Center, Ludwig Maximilians University Munich, Munich, Germany; 7 Department of Experimental Medical Science, Lung Bioengineering and Regeneration, Lund University, Lund, Sweden; Politecnico di Milano, ITALY

## Abstract

Allogeneic lung transplant is limited both by the shortage of available donor lungs and by the lack of suitable long-term lung assist devices to bridge patients to lung transplantation. Avian lungs have different structure and mechanics resulting in more efficient gas exchange than mammalian lungs. Decellularized avian lungs, recellularized with human lung cells, could therefore provide a powerful novel gas exchange unit for potential use in pulmonary therapeutics. To initially assess this in both small and large avian lung models, chicken (*Gallus gallus domesticus*) and emu (*Dromaius novaehollandiae*) lungs were decellularized using modifications of a detergent-based protocol, previously utilized with mammalian lungs. Light and electron microscopy, vascular and airway resistance, quantitation and gel analyses of residual DNA, and immunohistochemical and mass spectrometric analyses of remaining extracellular matrix (ECM) proteins demonstrated maintenance of lung structure, minimal residual DNA, and retention of major ECM proteins in the decellularized scaffolds. Seeding with human bronchial epithelial cells, human pulmonary vascular endothelial cells, human mesenchymal stromal cells, and human lung fibroblasts demonstrated initial cell attachment on decellularized avian lungs and growth over a 7-day period. These initial studies demonstrate that decellularized avian lungs may be a feasible approach for generating functional lung tissue for clinical therapeutics.

## Introduction

Allogeneic lung transplant remains the final available treatment modality and potentially life-saving intervention for patients with end-stage lung diseases. However, lung transplantation remains limited by a shortage of suitable donor lungs and many patients with end-stage lung diseases will succumb while on transplant waiting lists [1]. Further, there are few available bridging devices, comparable to left ventricular assist devices used in end-stage cardiac disease patients, for use in end-stage lung disease patients [2]. Extracorporeal membrane oxygenation (ECMO) devices have a significant role in short term acute neonatal respiratory diseases and a more limited role in acute adult respiratory diseases. However, ECMO requires hospitalization in critical care units and specialized health care providers [3, 4]. As such, it is not a practical or cost effective option for long-term bridging to lung transplant or as long-term support for end-stage lung disease patients who do not qualify for transplantation [2]. New innovative, cost-effective, and easily implementable technologies are desperately needed.

We and others have extensively studied the possibility of *ex vivo* lung bioengineering with a focus on de- and recellularization of mammalian lungs [5–22]. This includes developing potential transplantation strategies creating *ex vivo* autologous lungs from decellularized cadaveric or failed donor lungs recellularized with cells obtained from the eventual transplant recipient. This is a rapidly evolving, and promising approach although has not yet reached fruition. As opposed to mammalian lungs, avian lungs are static, multilayered structures in which gas exchange occurs by cross current exchange and is more efficient than the more complex mammalian lungs [23–26]. Notably, bird lungs have evolved to accommodate high metabolic needs required for flight by separating gas exchange from inflation/deflation, utilizing unidirectional, continuous air flow in the lung and a thinner blood-gas barrier. As such, avian lungs could provide potentially novel and effective bioscaffolds for use as lung assist devices and possibly also in transplantation schemes. We therefore postulate that decellularized avian lungs, recellularized with human lung epithelial, endothelial, and stromal cells, and/or with relevant stem and progenitor cells, including induced pluripotent stem cells, will provide a novel and more powerful gas exchange unit than recellularized mammalian lungs.

## Materials and methods

### Avian lungs

Chicken (*Gallus gallus domesticus)* and emu (*Dromaius novaehollandiae*) lungs were procured *post mortem* from local farms/slaughterhouses. Healthy chickens were euthanized via a standard method of slitting the throat and exsanguination at a local farm, and the carcass was immediately cooled on ice and transported to the laboratory for dissection. Heart-lung-trachea blocs were harvested via bilateral dorsal thoracotomy approach: the trachea was initially identified and isolated in the neck, separated from the esophagus, and cannulated with a tubing connector (inner diameter 5/32” inch and a Luer lock female connector, Cole-Parmer) which was secured in place using silk suture. The dorsum of the chicken was identified and the bilateral scapulae were mobilized superficially from the rib cage below. The thorax of the bird was then entered bilaterally rostral to the lung, with care not to damage underlying structures. The lungs and air sacs were identified and separated. Using blunt dissection, the lungs were carefully peeled from the costal structures, leaving the air sacs *in situ*. Anteriorly, the trachea was followed rostrally until the heart and lung structures were identified. The trachea, heart, and lungs were then separated and removed *en bloc*. The pulmonary arteries were identified bilaterally and preserved, as was the entirety of the tracheobronchial tree. If upon perfusing of the trachea an air sac ostium was identified, it was ligated with a small surgical clip or suture. The pulmonary arteries were each identified and individually cannulated with blunted 18-gauge needles bilaterally.

Healthy emus being processed for commercial use were euthanized under standard protocol at a local farm. Lungs were identified, isolated, and preserved by the farm personnel in collaboration with the research team. Samples were preserved on ice and transported to the laboratory for immediate decellularization. Emu lungs were cannulated via their main bronchus and pulmonary arteries in a similar fashion as the chickens. Given the larger size of the emu lungs, two approaches were utilized: in some cases, both lungs were processed together, in others, the right and left lungs were separated and processed individually. All experimental protocols were approved by the Institutional Animal Care and Use Committee of the University of Vermont, in agreement with local and national laws and regulations.

### Injection molding

Two chickens were euthanized and their tracheae were cannulated. The rest of the animal was left intact in order to not disturb and prevent rupture of air sacs. Oomoo® 30 silicone solution (Smooth-On, Inc., Macungie, PA, USA) was utilized as per manufacturer’s instructions. In order to reduce its viscosity, kerosene 1-K Heater Fuel (Klean Strip, Memphis, TN, USA) was added at a 1:8 ratio to the silicone solution. Using a Luer lock 60 mL syringe, the silicone was injected via the cannulated trachea into the native tissue. The chicken was held vertically, so that the trachea was the highest point in the system, allowing air to escape during the silicone instillation. Once the airways were filled with 300 mL of silicone, resembling the approximate volume of the chicken airway [27], the syringe was left locked to the trachea to maintain inflation pressure. After overnight silicone curation, tissue was removed mechanically using surgical tools. The mold was then submerged in a 1 M hydrogen sulfate [HSO_4_^-^] solution overnight. If tissue had not cleared, the solution was replaced and the mold was submerged again overnight. After the isolation was complete, the mold was submerged in 70% ethanol overnight for disinfection.

### Lung decellularization

The decellularization protocol was slightly modified from the one we utilize in mammalian lungs, including human lungs [5, 10, 12, 16]. After harvesting and anatomic identification of major artery and airways (either trachea or bronchi), each lung was thoroughly perfused through the airway and vasculature with deionized water (DI) containing heparin sulfate (1U/ml, Fisher Scientific, Waltham, MA, USA) to clear all blood. Each lung (for emus) or set of lungs (for chickens) was perfused with the following detergents and membrane-destabilizing solutions in a sequentially fashioned protocol using a roller pump (Stockert Shiley) [5]. Under sterile conditions, each lung was perfused with 4L of DI solution, followed by 4L of 0.1% Triton X-100 (Sigma-Aldrich, St. Louis, MO, USA) solution and kept in 0.1% Triton X-100 for 24 hours at 4°C on a shaker. After incubation, each lung was perfused with 4L DI, followed by 4L of 2% sodium deoxycholate (SDC, Sigma-Aldrich) solution and kept in 2% SDC for 24 hours at 4°C on a shaker. Lungs were again perfused with 4L DI, followed by 4L of 1 M sodium chloride (NaCl, Sigma-Aldrich) and kept in 1M NaCl solution for 1 hour at room temperature (RT) on a shaker. Then, each lung was rinsed with 4L DI followed by perfusion of 4L DNAse solution (30 mg/L bovine pancreatic deoxyribonuclease, 1.3 mM MgSO_4_, 2 mM CaCl_2_ in DI water, all Sigma Aldrich). Lungs were kept in DNAse solution for 1 hour at RT on a shaker. After again perfusing with 4L DI, lungs were perfused with 4L 0.1% peracetic acid (Sigma-Aldrich) in 4% ethanol per lung and kept in 0.1% peracetic acid solution at RT for 1 hour on a shaker. Afterwards, lungs were perfused with storage solution, which contains 1x PBS solution (Corning, Corning, NY, USA) supplemented with Penicillin/Streptomycin (500 IU/mL Penicillin/500 μg/mL Streptomycin, Lonza, Basel, Switzerland), Gentamicin (50 mg/L, Corning), and Amphotericin B (2.5 mg/L, Corning). At the conclusion of the decellularization protocol, biopsies were procured and the samples were stored in storage solution at 4°C until further processing or usage for reseeding. Lungs were stored for a maximum of 3 months based on previously determined optimal storage duration for decellularized mammalian lungs [11].

In comparison to mammalian lungs, bird lungs work through a cross current system. As such, each step of the perfusion decellularization was performed utilizing a continuous loop perfusion pump (as opposed to intermittent filling) to maximize lung filling and detergent efficacy. This was accompanied by intermittent gently tissue compression after each filling, similar to the procedure used for the decellularization of mammalian lungs [28,29]. Perfusion was performed at a rate of 2 L/min for chicken and 3 L/min for emu lungs for a total of 10 minutes on-pump with recirculation of the respective solution. These flow rates were designed to maintain a full, static volume within the lung, with distention. At completion of on-pump perfusion the organs were transferred to their respective solutions on a shaker for further decellularization.

### Measurements of flow resistance

After excision, the main parabronchi and pulmonary artery were cannulated in 3 individual chicken lobes. The cannulated pulmonary artery and parabronchi were connected alternately to a controlled perfusion system with continuous pressure (gravimetric level) at 25 cmH_2_O. The flow rate was obtained as the relation after collecting the volume of 1x PBS that was perfused during 30 seconds through each way (vasculature or airway). Vascular resistance (R_v_) and airway resistance (R_airway_) were calculated by dividing the flow rate by the continuous pressure.

### Assessment of residual DNA

Native and decellularized lung tissue was dried on a tissue paper (Kimwipe, Kimtech, Kimberly-Clark, Roswell, GA, USA) until no liquid was visibly seen to be released from it, weighed, and DNA was extracted using the DNeasy Blood & Tissue Kit (Qiagen, Hilden, Germany) following the instructions provided by the manufacturer. The isolated DNA was run on a 0.8% agarose gel and visualized under UV light with SYBR Safe DNA Gel stain (Invitrogen, Carlsbad, CA, USA) using the Versa Doc (BioRad, Hercules, CA, USA). A 100 bp ladder and salmon sperm DNA (Invitrogen) was used as DNA size marker and positive control. DNA was quantified using a Nanodrop (Thermo Scientific) and threshold for adequate decellularization was set at less than 50 ng/mg dry tissue weight [30].

### Assessment of residual detergent

Concentrations of SDC in wash effluents were determined using a modified methylene blue (MB) assay previously utilized in decellularized mammalian lungs [15]. In short: effluent samples were mixed with 0.0125% MB (Sigma-Aldrich) in DI water (w/v) at a ratio of 1:10. After vortexing the samples with MB, chloroform (Sigma-Aldrich) was added at a ratio of 1:2 (sample: chloroform, v/v). Samples were then vortexed for 1 min. Following a 30-minute incubation period at RT, 150 μl of the bottom chloroform layer was extracted and the absorbance at 630 nanometers (nm) was measured in a Synergy HT Multi-Detection Microplate Reader (Biotek Instruments, Winooski, VT, USA) in a polypropylene 96-well plate (Costar, Corning, NY, USA). Pure DI-water or PBS (Mediatech Inc., Manassas, VA, USA) containing no detergents served as the blank. SDC concentration was calculated based on SDC standard curves prepared in either DI water (for Triton, SDC, NaCl, and DNAse effluents) or storage solution (for PBS effluents).

### Lung histology

Decellularized lung slices were fixed in 4% paraformaldehyde by immersion overnight at room temperature and no inflation pressure was utilized during the fixation. Then slices were embedded in paraffin, and 5-μm sections mounted on glass slides. Following deparaffinization, sections were stained with hematoxylin & eosin, Verhoeff’s Van Giesson (EVG), Masson’s Trichrome, or Alcian Blue, and were assessed by brightfield light microscopy. Alizarin red was used for histological assessment of possible hMSC differentiation in recellularized chicken and emu lungs.

### Electron microscopy

For electron microscopic analyses, segments of decellularized chicken and emu lungs were fixed overnight at 4°C in Karnovsky’s fixative (2.5% glutaraldehyde, 1.0% paraformaldehyde in 0.1M Cacodylate buffer, pH 7.2). After rinsing in Cacodylate buffer, the tissue was minced into 1 mm^3^ pieces and then fixed in 1% osmium tetroxide for 2 hours at 4°C. Subsequently, the pieces were rinsed again in Cacodylate buffer, dehydrated through graded ethanols, then cleared in propylene oxide, and embedded in Spurr’s epoxy resin (all reagents from Electron Microscopy Sciences, Hatfield, PA, USA). Semi-thin sections (1 μm) were cut with glass knives on a Reichert ultracut microtome (Reichert-Jung, Vienna, Austria), stained with methylene blue–azure II (Electron Microscopy Sciences) and then evaluated for areas of interest (proximal and distal alveolar septae, large/small airways, blood vessels). Ultrathin sections (60–80 nm) were cut with a diamond knife, retrieved onto 200 mesh thin bar nickel grids (Electron Microscopy Sciences), contrasted with uranyl acetate (2% in 50% ethanol, Electron Microscopy Sciences) and lead citrate (Electron Microscopy Sciences), and examined with a JEOL 1400 TEM (JEOL USA, Inc, Peabody, MA, USA) operating at 60kV [5].

### Immunohistochemical staining

Standard deparaffinization was performed with three separate 10 min incubations in xylenes (Fisher Scientific), followed by rehydration in a descending series of ethanols, and finally in water. Antigen retrieval was performed by heating tissue in 1x sodium citrate buffer (Dako, Carpentaria, CA, USA) at 98°C for 20 minutes followed by a brief 20 minutes cool at room temperature. Tissue sections were permeabilized in 0.1% Triton X-100 solution for 15 minutes. Triton X-100 was removed with two 10-minute washes in 1% BSA (Sigma) solution. Blocking was performed with 10% goat serum (Jackson Immuno Research, West Grove, PA, USA) for 60 minutes. After blocking, primary antibody was added and tissue sections were incubated overnight at 4°C in a humidified chamber. Tissues were washed three times with 1% BSA solution for 5 minutes each. Secondary antibody was added and incubated for 60 min at room temperature in a dark humidified chamber. Tissues were again washed three times in 1% BSA solution for 5 minutes each in the dark. DAPI nuclear stain (Invitrogen/Life Technologies/Thermo Fisher) was added for 5 minutes at room temperature in the dark followed by 2 washes in 1% BSA solution for 5 minutes each. The sections were finally mounted in Aqua Polymount (Lerner Laboratories, Pittsburg, PA, USA).

As there are limited antibodies available specifically against bird proteins, we utilized those available and when not available, utilized commercially available antibodies for mammalian proteins. As detailed in the results section, we were able to validate reactivity of each antibody utilized with the respective bird proteins. Primary antibodies used were: purified mouse anti-fibronectin monoclonal (610077–1:100 –BD Transduction Laboratories, Franklin Lakes, NJ, USA, reactivity with chicken confirmed in development), laminin antibody polyclonal (ab11575–1:100 –Abcam, Cambridge, United Kingdom, reactivity with bird unknown), rabbit polyclonal to alpha elastin (ab21607–1:100 –Abcam, reactivity with bird unknown), smooth muscle myosin heavy chain 2 polyclonal (ab53219–1:100 –Abcam, predicted to work with vertebrates), collagen I polyclonal (ab292–1:100 –Abcam, reactivity with bird unknown), Ki67 proliferation marker polyclonal (ab16667–1:50 –Abcam, reactivity with bird unknown), cleaved caspase- 3 polyclonal (Asp175–1:100 –Cell Signaling Technology, Danvers, MA, USA, reactivity with bird unknown), mouse clone anti-human actin polyclonal (1A4–1:10,000—Dako via FAHC, Denmark, antibody cross-reacts with the α-smooth muscle actin-equivalent protein in chicken). For the identification of discriminant phenotypic variations of the different cell types used for the recellularization of chicken and emu lungs the following biomarkers were utilized: Anti-E-Cadherin (610181–1:100 -BD), anti-PECAM1 (HPA004690–1:100—Sigma), mouse anti-human Actin (1:10000—DAKO) and Thy1 (CD90) (13801–1:100—Cell Signaling) for HBE [31], CBF [32], HLF [33] and hMSC [34], respectively. Secondary antibodies used: Alexa Fluor 568 goat anti-rabbit IgG (H+L) (1:500, Invitrogen), Alexa Fluor 568 F(ab’)2 fragment of goat anti-mouse IgG (H+L) (1:500, Invitrogen) [5, 8–10, 12]. Additionally, a semi-quantitative analysis of the mean fluorescence intensity of collagen I, collagen IV and elastin proteins in chicken and emu lung slices were performed using ImageJ.

### Mass spectrometry

Three samples of about 125 mg wet weight from different locations of each decellularized lung tissue were procured. Each sample was homogenized using a Polytron PT2100 (Kinematica, Luzern, Switzerland) in 200 μl of 4x lysis buffer (250 mM Tris pH 6.8, Sigma, 8% SDS, BioRad, 400mM DTT, Sigma, 40% Glycerol, Sigma) and diluted with DI water to 1x. After centrifugation for 5 min at 15,000 g at 4°C supernatant and pellet were separated. Protein content of the supernatant was evaluated with the DC detergent compatible protein detection Kit (BioRad). 20 μg of protein were loaded onto a 10% SDS PAGE gel and individual bands containing chicken or emu proteins were excised and prepared for mass spectrometry using a standard in-gel trypsin digestion protocol as described previously [35]. Briefly, gel bands were cut into 1 mm^3^ pieces and destained overnight using 50 mM ammonium bicarbonate in 50% acetonitrile. After reduction by 10 mM dithiothreitol (DTT) at 55°C for 1 hour the gel pieces were alkylated with 55mM iodoacetamide (IAA) in the dark at room temperature for 45 min. The gel pieces were then washed and dehydrated twice alternately with 100 mM ammonium bicarbonate and 100% acetonitrile (ACN). The gel pieces were dried in a SpeedVac (Thermo Savant, Waltham, MA, USA) and then subjected to trypsin digestion using sequencing grade trypsin (Promega, Madison,WI, USA) for 17 hours at 37°C. The tryptic digests were acidified with 150 μl of 5% formic acid (FA) in 50% acetonitrile to stop the reaction. The peptides were extracted, dried and kept in a -80°C freezer until they were analyzed by mass spectrometry.

The dried digests were re-suspended in 50 μl of 2.5% ACN / 2.5% FA in water and 5 μl of sample was loaded onto a capillary fused silica column (12 cm x 100 μm inner diameter) packed with HALO C18 (2.7 μm particle size, 90 A, Michrom Bioresources, CA, USA) and run at a flow rate of 300 nL/min. Peptides were separated by a gradient of 0–35% ACN/0.1%FA (Fisher Chemical, Optima, LC/MS grade) over 120 min, 35–100% ACN /0.1% FA for 1 min, and a hold of 100% ACN for 8 min, followed by an equilibration 0.1% FA in H_2_O for 21 min. Peptides were introduced to the Q-Exactive mass spectrometer (Thermo Fisher Scientific, Waltham, MA, USA) via a nanospray ionization source and a laser pulled ~3 μm orifice with a spray voltage of 2.2 kV. Mass spectrometry data were acquired in a data-dependent “Top 10” acquisition mode, in which a survey scan from *m/z* 360–1600 was followed by 10 higher -energy collisional dissociation (HCD) tandem mass spectrometry (MS/MS) scans of the most abundant ions. MS/MS scans were acquired with the following parameters: isolation width = 1.6 *m/z*, normalized collision energy = 26.

Product ion spectra were searched using the SEQUEST HT engine implemented on the Proteome Discoverer 1.4 (Thermo Fisher Scientific, Waltham, MA, USA) against a Uniprot *Gallus gallus* (UP000000539, May 14, 2016 release was downloaded) *and Dromaius novaehollandiae* from the NCBI protein database (May 14, 2016). Search parameters were as follows: (1) full trypsin enzymatic activity, (2) two missed cleavages, (3) peptides between the MW of 350–5000, (4) mass tolerance at 20 ppm for precursor ions and 0.02 Da for fragment ions, (5) Dynamic modifications on methionine (+15.9949 Da: oxidation), (6) 3 maximum dynamic modifications allowed per peptide; and (7) static modification on cysteine (+57.0215 Da: carbamidomethylation). The combined data set was filtered to contain less than 1% false positive (with the Target Decoy PSM Validator node).

Proteins positively identified with two or more distinct peptide hits were assigned to one of six groups: ECM, cytoplasm, cytoskeletal, nuclear, membrane-associated, secreted, and uncharacterized in case no subcellular location was specified. Heatmaps were generated with the log2 transformation of peptide hits from each positively identified protein with clustering of the rows to display genes that are similarly expressed [5, 7, 10].

### Preparation and culture of recellularized chicken lungs and emu segments

Whole chicken lungs or small, approximately 10–15 cm^3^ pieces of decellularized emu lungs excised from the larger lobes were used. Under sterile technique, the largest corresponding bronchus or parabronchus of the lung/segment was cannulated with blunted 18.5 or 25G cannulas. After the cannulas were secured with titanium clips (Teleflex Medical, Wayne, PA, USA) the lungs/segments were coated in 2.5% sodium alginate (Manugel, FMC Biopolymer, Philadelphia, PA, USA) and then immediately cross-linked with a 3% calcium chloride (Sigma) solution, resulting in segments being uniformly coated in a calcium alginate hydrogel that serves as an artificial pleural coating [5]. Hydrogel-coated lungs/segments were then inoculated with cell suspensions (4.5–5.0 x10^7^ cells per lung/segment, suspended in 1.0 ml media) in the respective compartment (Human bone marrow-derived mesenchymal stromal cells (hMSCs), Human Lung Fibroblasts (HLF), Human bronchial epithelial cells (HBE) via the airway and pulmonary endothelial colony forming cells (CBF) via the vasculature) and allowed to incubate in cell cultivation medium at 37°C overnight to allow cellular attachment. The following day lungs/segments were sliced into approximately 1 mm thin sections with sterile razor blades and each slice placed in an individual well of a 24-well non-tissue culture treated dish, covered with 2 mL of sterile cell cultivation media, and placed in a standard tissue culture incubator at 37°C with 5% CO^2^ as previously described [5]. Cell cultivation media was replaced routinely at 48-hour intervals. Slices were harvested at 1, 3, 7, 14, and 28 days post-inoculation and fixed for at least 4 hours at room temperature in 4% paraformaldehyde. Harvested samples were embedded in paraffin, cut, and mounted as 5 μm sections, and then assessed by H&E staining for the presence and distribution of the inoculated cells.

### Cells and seedings

Cells representative for the main types of cells residing inside the lung tissue and of interest for organ regeneration were used. Human bronchial epithelial (HBE) cells (courtesy of Albert van der Vliet, University of Vermont, originally from Dr. J. Yankaskas [36] were cultured on cell-culture treated plastic at 37°C and 5% CO^2^ in serum free culture medium consisting of DMEM/F-12 50/50 mix (Corning), 10 ng/ml cholera toxin (Sigma), 10 ng/ml epidermal growth factor (Sigma), 5 μg/ml insulin (Gemini Bio-Products, West Sacramento, CA, USA), 5 μg/ml transferrin (Sigma), 0.1 μM dexamethasone (Sigma), 15 μg/ml bovine pituitary extract (Sigma), 0.5 mg/ml bovine serum albumin (Life Technologies), and 100 IU/ml penicillin/100 μg/ml streptomycin (Corning). HLF (ATCC, CCL 171) were grown in media consisting of DMEM/F-12 50/50 mix (Corning), 10% fetal bovine serum (Hyclone), 100 IU/ml penicillin/100 μg/ml streptomycin, 2mM L-glutamine (Corning). CBF cells were obtained from Mervin Yoder (Indiana University–Purdue University Indianapolis) and grown in EGM-2 (Lonza) supplemented with 5% fetal bovine serum, 0.04% hydrocortisone, 0.4% hFGF-B, 0.1% VEGF, 0.1% R3-IGF-1, 0.1% ascorbic acid, 0.1% hEGF, 0.1% gentamicin sulfate Amphotericin-B, and 100 IU/ml penicillin/100 μg/ml streptomycin. These cells were expanded on collagen type I coated tissue culture surfaces. hMSCs were obtained from the University of Minnesota through the NHBLI Production Assistance for Cell Therapy program. These cells have previously been extensively characterized for cell-surface marker expression and differentiation capacity [37]. Cells were expanded in culture using media consisting of Modification of Eagle Medium-Earls Balanced Salt Solution (MEM-EBSS) (Hyclone, Thermo Scientific), 20% fetal bovine serum, 100 IU/ml penicillin/100 μg/ml streptomycin, 2mM L-glutamine, and used only at no more than passage 7.

### Statistical analyses

Flow resistances were compared using paired t-test. For mass spectrometry assessments, heatmaps for the log2 of unique peptide hits for each positively identified protein in the mass spectrometric analyses of lungs decellularized under each experimental condition were generated using the 'pheatmap' package for 'R' statistical software version 2.15.1 [5, 7, 10, 11, 13]. Differences between Ki67 or caspase-3 expression were assessed by two-way ANOVA with Bonferroni post-test. Significance (*) was determined by p<0.05. Comparisons between fluorescence intensity were performed by paired t-test.

## Results

### Decellularized avian lungs qualitatively maintain extracellular matrix structure by histologic and immunohistochemical evaluations with maintenance of key extracellular matrix proteins similarly present in decellularized mammalian lungs

**Fig 1:**
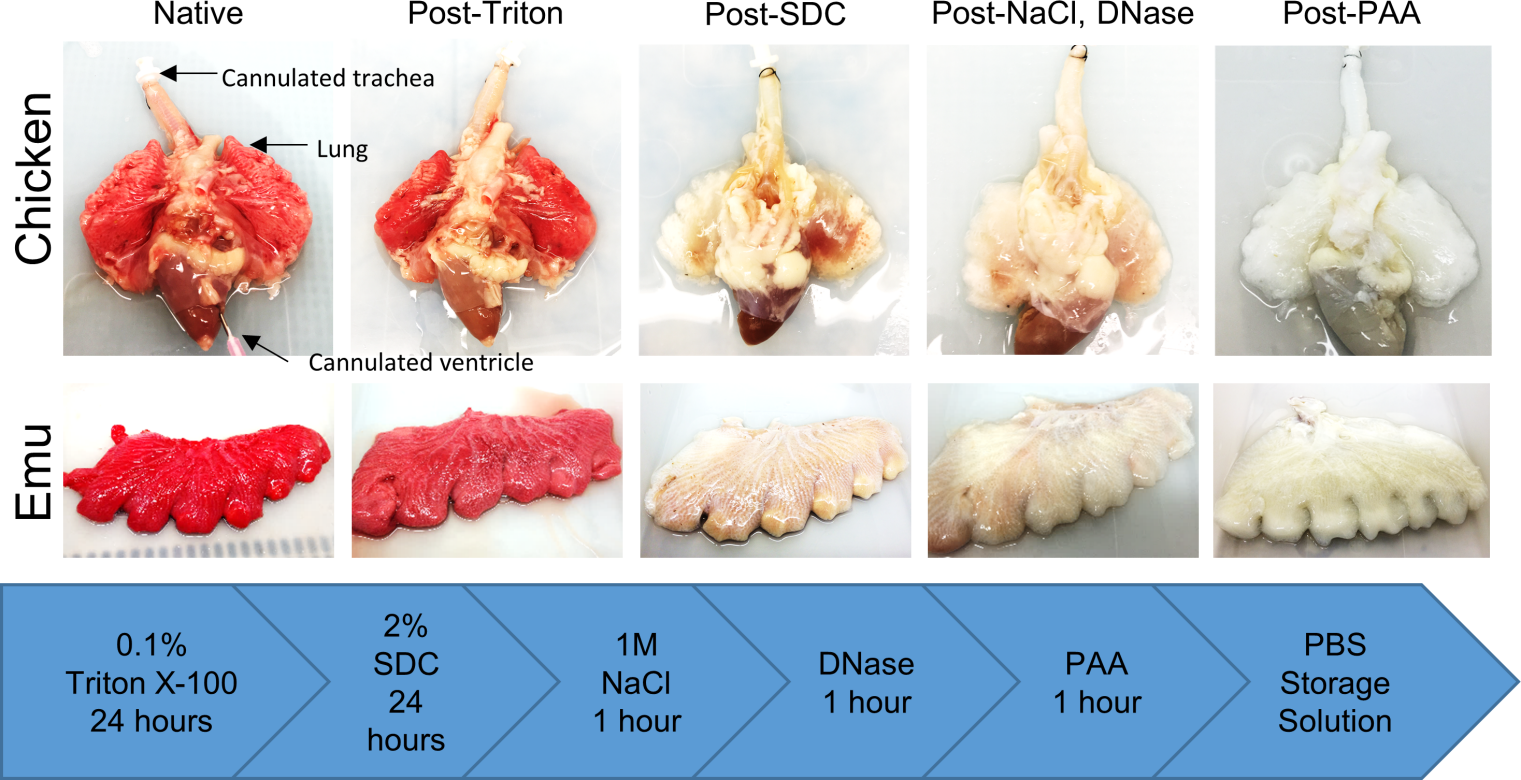
Chicken and emu bird lungs are comparably grossly decellularized. Progressive decellularization results in comparable clearing of blood and pink coloration resulting in final pearly white translucent tissues. Representative images from chicken (n = 14) and emu (n = 7) bird lungs are shown. SDC = sodium deoxycholate, NaCl = sodium chloride, DNase = DNase solution, PAA = peracetic acid, PBS = phosphate buffered saline.

**Fig 2:**
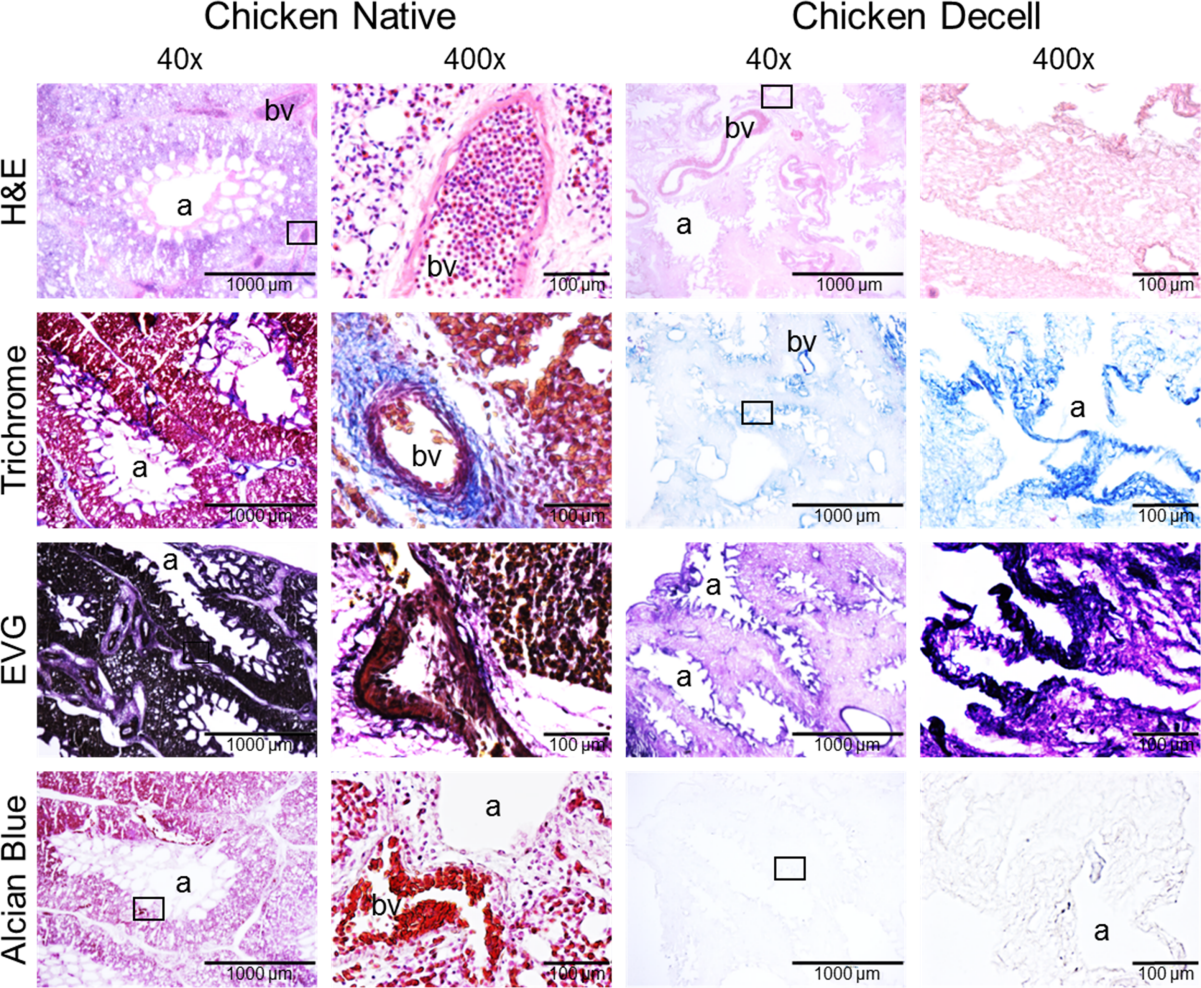
The decellularization process largely preserves the native structure of chicken lungs. Representative images of native and decellularized chicken lungs are depicted. Photomicrographs demonstrate qualitative preservation of characteristic structure and major ECM proteins (collagen, elastin) by H&E, EVG, and trichrome stains. Glycosaminoglycan content is qualitatively decreased as assessed by Alcian blue staining. a = airways, bv = blood vessels. Representative images from chicken lungs (n = 14) are shown. Original magnification 40X and 400X, scale bar is indicated on each image.

**Fig 3:**
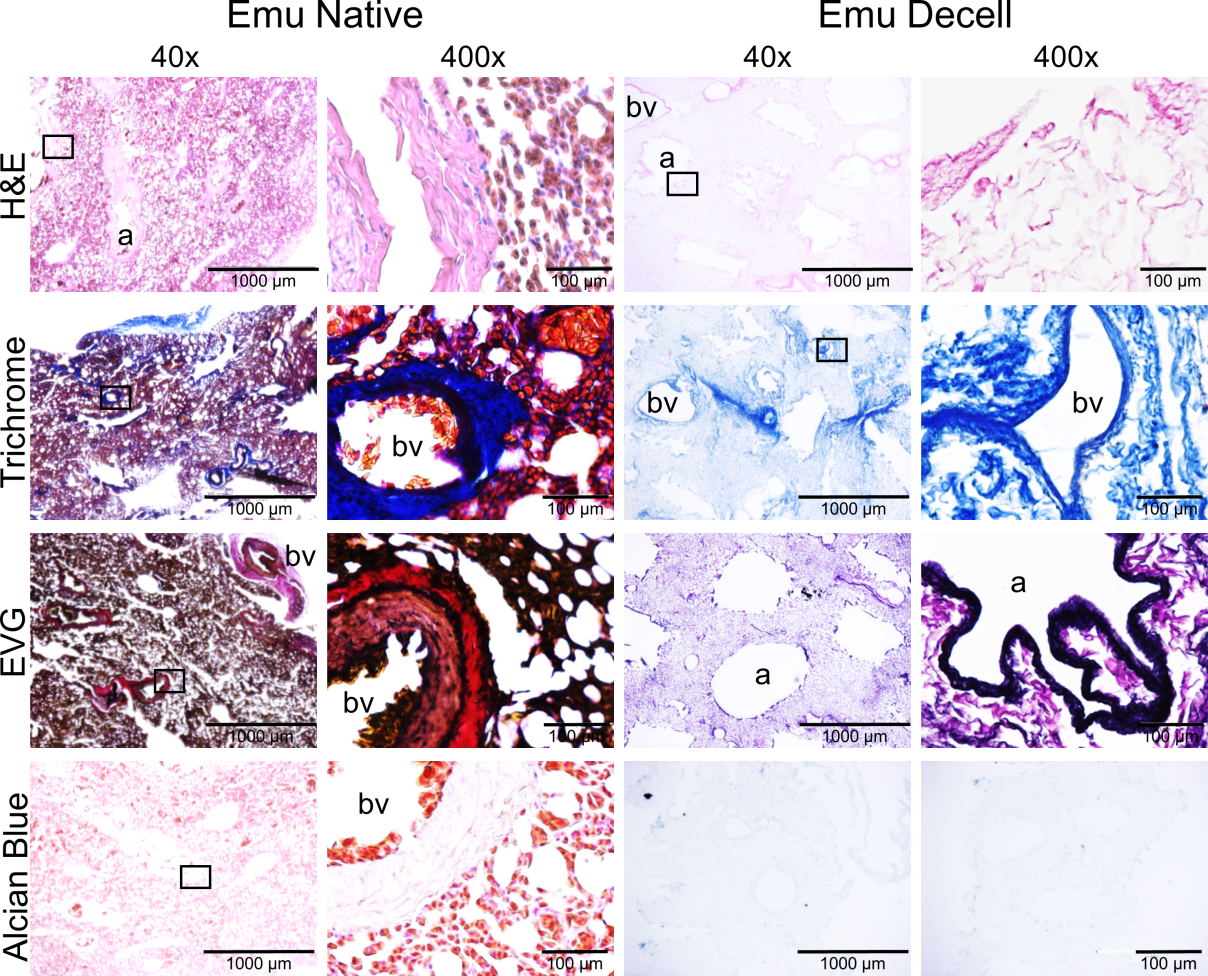
The decellularization process largely preserves the native structure of emu lungs. Representative images of native and decellularized emu lungs are depicted. Photomicrographs demonstrate qualitative preservation of characteristic structure and major ECM proteins (collagen, elastin) by H&E, EVG, and trichrome stains. Glycosaminoglycan content is qualitatively decreased as assessed by Alcian blue staining. a = airways, bv = blood vessels. Representative images from emu lungs (n = 4) are shown. Original magnification 40X and 400X, scale bar is indicated on each image.

**Fig 4:**
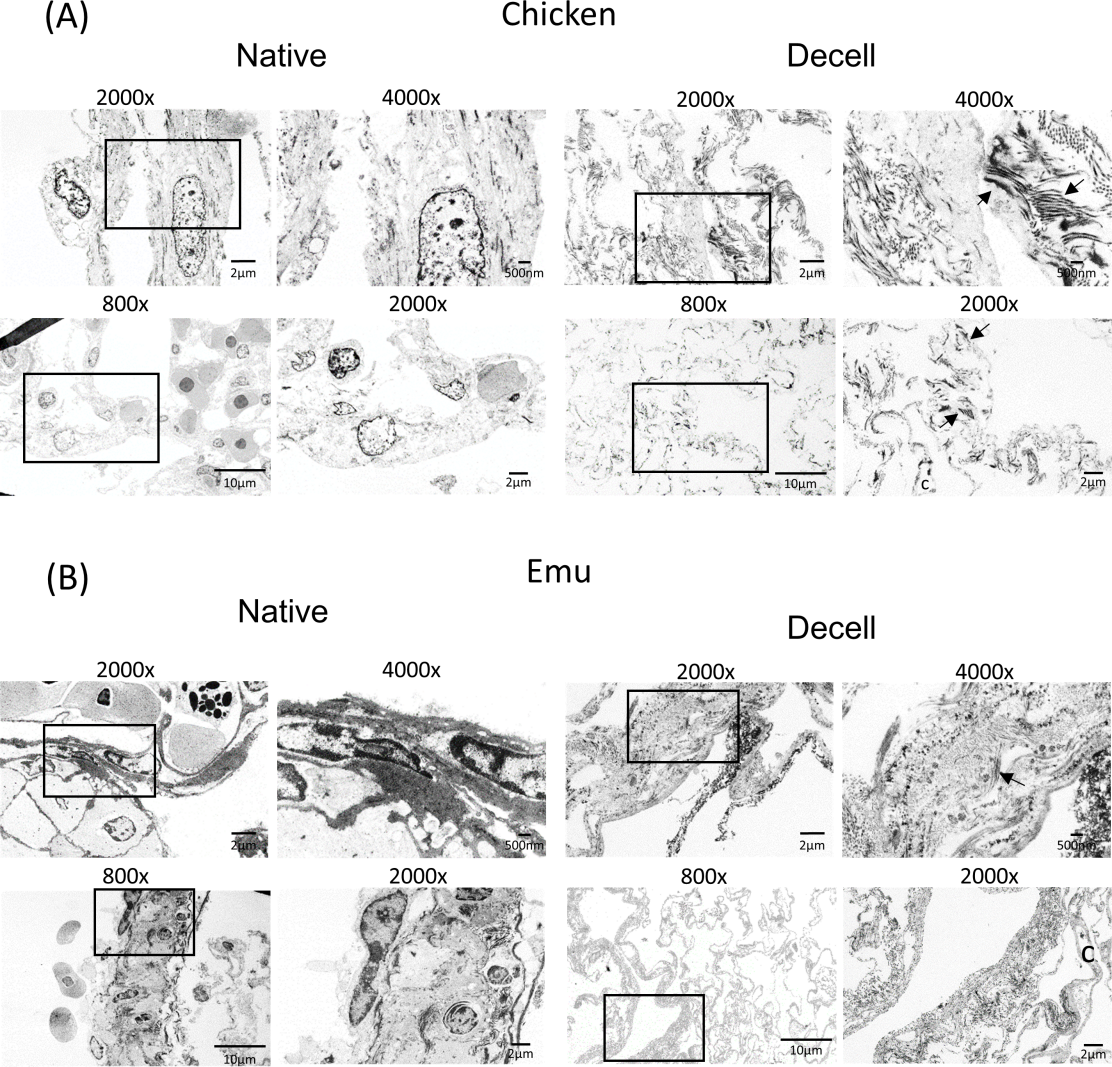
The decellularization process largely preserves the ultrastructure of bird lung extracellular matrix. Transmission electron microscopy demonstrates comparable appearance of the parabronchial microstructures in decellularized (A) chicken and (B) emu bird lungs. Representative images from a single decellularized chicken and single emu bird lung are shown. Enlargements of the inserts for each image demonstrate more structural details. Collagen fibers are indicated by arrows and capillaries are indicated by “c”. Original magnification and scale bar is indicated on each image.

**Fig 5:**
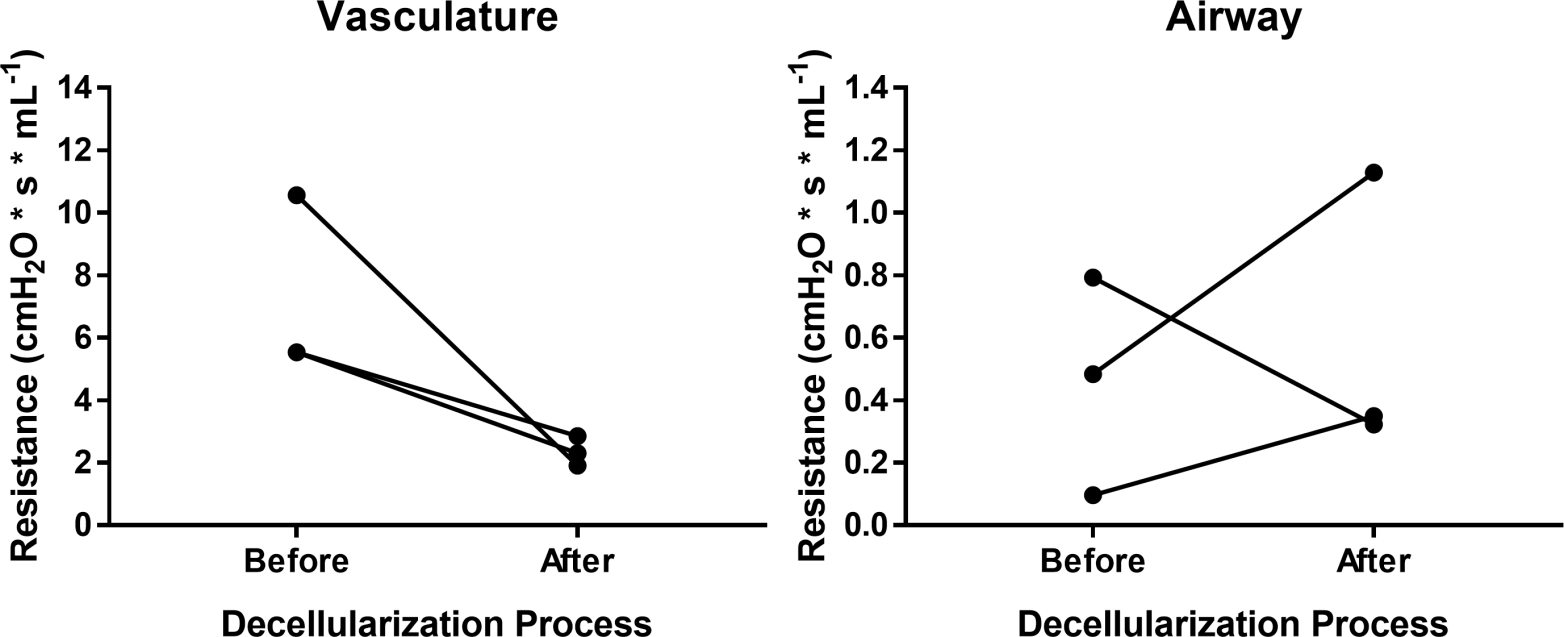
Flow resistances measurements showed no significant alterations after decellularization. Vascular resistance (R_v_) and airway resistance (R_airway_) of whole chicken lungs were assessed before and after completion of the decellularization process.

**Fig 6:**
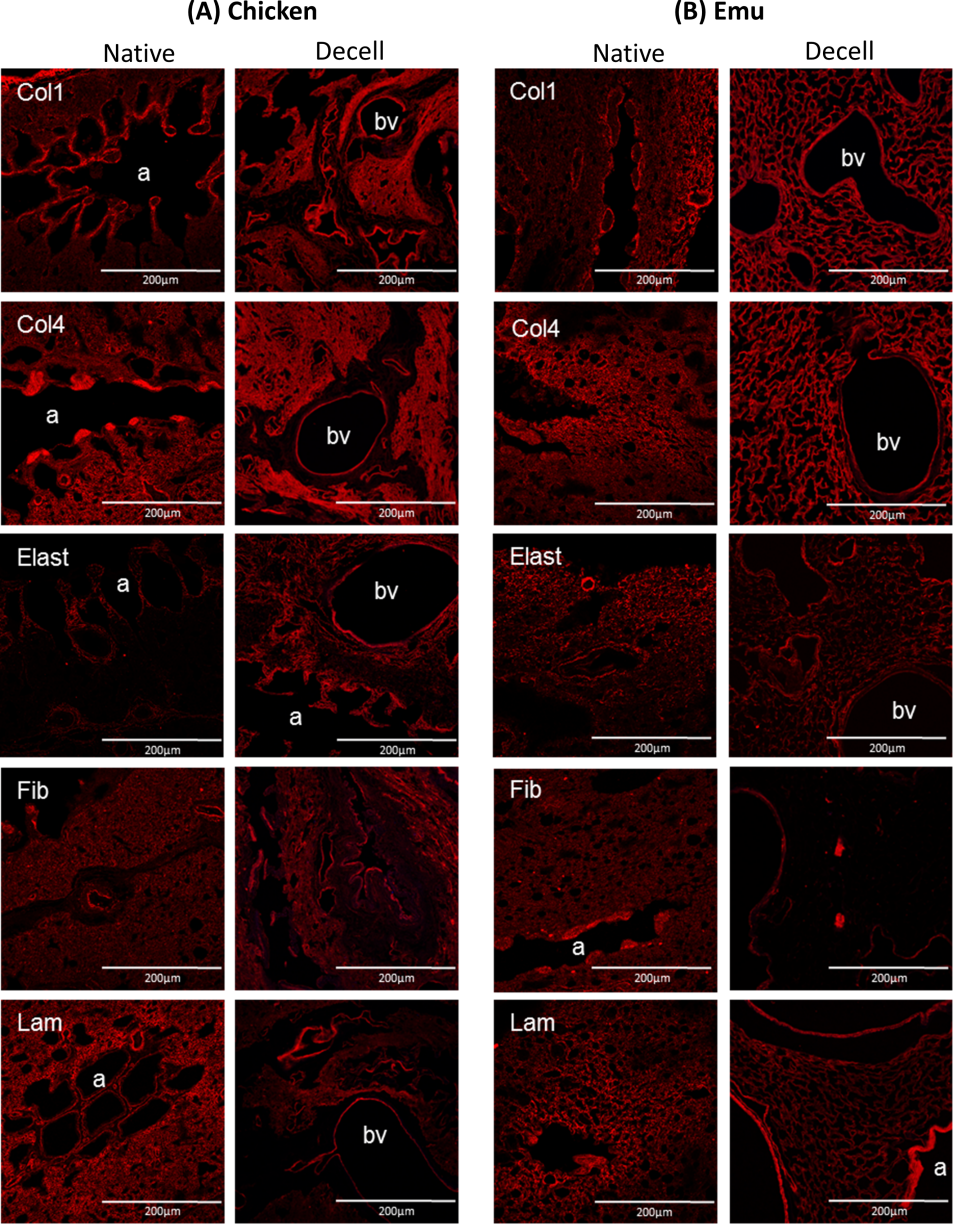
Decellularization preserves major ECM proteins in chicken and emu bird lungs. Representative photomicrographs comparing native and decellularized (A) chicken and (B) emu bird lungs are depicted and demonstrate similar qualitative retention of major structural ECM proteins. Stain of interest is depicted in red. Col1 = type I collagen, Col4 = type 4 collagen, Elast = elastin, Fib = fibronectin, Lam = laminin, bv = blood vessel, a = airway. Original magnifications 200X, scale bar 200μm. a = airways, bv = blood vessels. Representative images from 2 chicken and 2 emu bird lungs are shown.

A Triton X-100/sodium deoxycholate (SDC) detergent-based decellularization protocol with constant flow perfusion (2 liters/minute) of both the vasculature and airways, a method previously optimized for use in mammalian lungs, was adapted for use in avian lungs. Chicken and emu lungs underwent successful decellularization as demonstrated by the progressive loss of pink coloration leading to a final translucent pearly white gross appearance. Greater understanding of avian pulmonary anatomy of the decellularized lungs was gained by creating the injection molds of native chicken lungs (S1 Fig). This allowed for more effective identification and ligation of air sacs prior to decellularization (Fig 1). Overall, the microarchitecture of the lungs was preserved as observed in histologic stainings with hematoxylin and eosin (H&E), Verhoeff’s Van Gieson (EVG), Masson’s trichrome, and Alcian blue (Figs 2 and 3). There was no residual cellular debris or cellular material detectable in either chicken (Fig 2) or emu (Fig 3) lungs. Similar to the decellularization of mammalian lungs (rodent, porcine, non-human primate, or human), a qualitative decrease in both elastin and glycosaminoglycans was observed with EVG and Alcian blue staining, respectively. Electron microscopic evaluation demonstrated retention of characteristic ECM structures including collagen fibrils and intact capillaries (Fig 4). Vascular resistance and airway resistance showed no significant alterations after completion of the decellularization process (Fig 5). DNA quantification in decellularized chicken and emu lungs demonstrated residual levels below 50 ng/mg and no residual fragments (200 basepairs or less) were observed on DNA gels, thereby suggesting adequate removal of nuclear material (S2 Fig) [38]. Immunofluorescence staining for specific ECM proteins (Fig 6) demonstrated general qualitative retention of collagen I, collagen IV, and laminin, in both chicken and emu lungs. After performing a semi-quantitative analysis of the mean fluorescence intensity (S3 Fig) elastin and fibronectin seemed decreased in emu lungs only (Fig 6). Notably, as many of the commercial antibodies utilized had not been previously validated in chicken and emu tissue, appropriate positive and negative controls demonstrated cross-reactivity with the bird lung ECM proteins (S4 Fig). Furthermore, it is known that residual detergent in the decellularized lungs will negatively affect cell viability and proliferation. Therefore, residual detergent (SDC) was assessed and was found to be below detectable or significant limits in both decellularized chicken and emu lungs (S5 Fig)

### Mass spectrometry analysis of decellularized chicken and emu lungs is limited by the available databases

**Fig 7:**
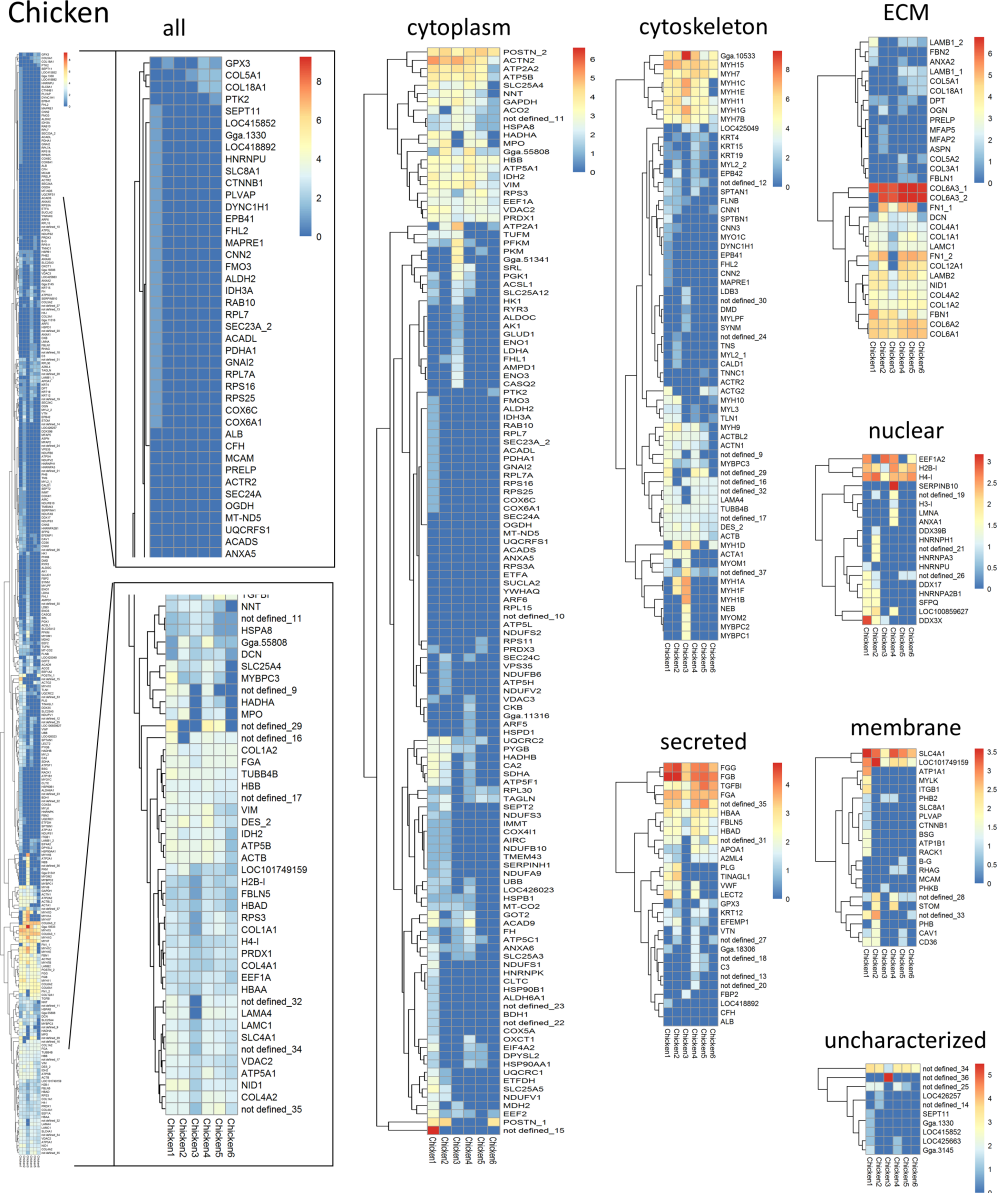
Mass spectrometric assessment of residual proteins following decellularization of chicken lungs demonstrates overall concordance in residual proteins detected. Positively identified proteins in decellularized chicken lungs (i.e. those proteins which were detected with at least 2 unique peptide hits and exceeded the FDR cutoff for identification) were assigned to groups according to subcellular location (cytoskeletal, cytosolic, ECM, membrane-associated, nuclear, secreted, and uncharacterized in case no subcellular location was specified). Heatmaps were generated using the log2 transformation of total peptide counts for all positively identified proteins and grouped by category. Representative heatmaps from 6 chicken lungs are shown.

**Fig 8:**
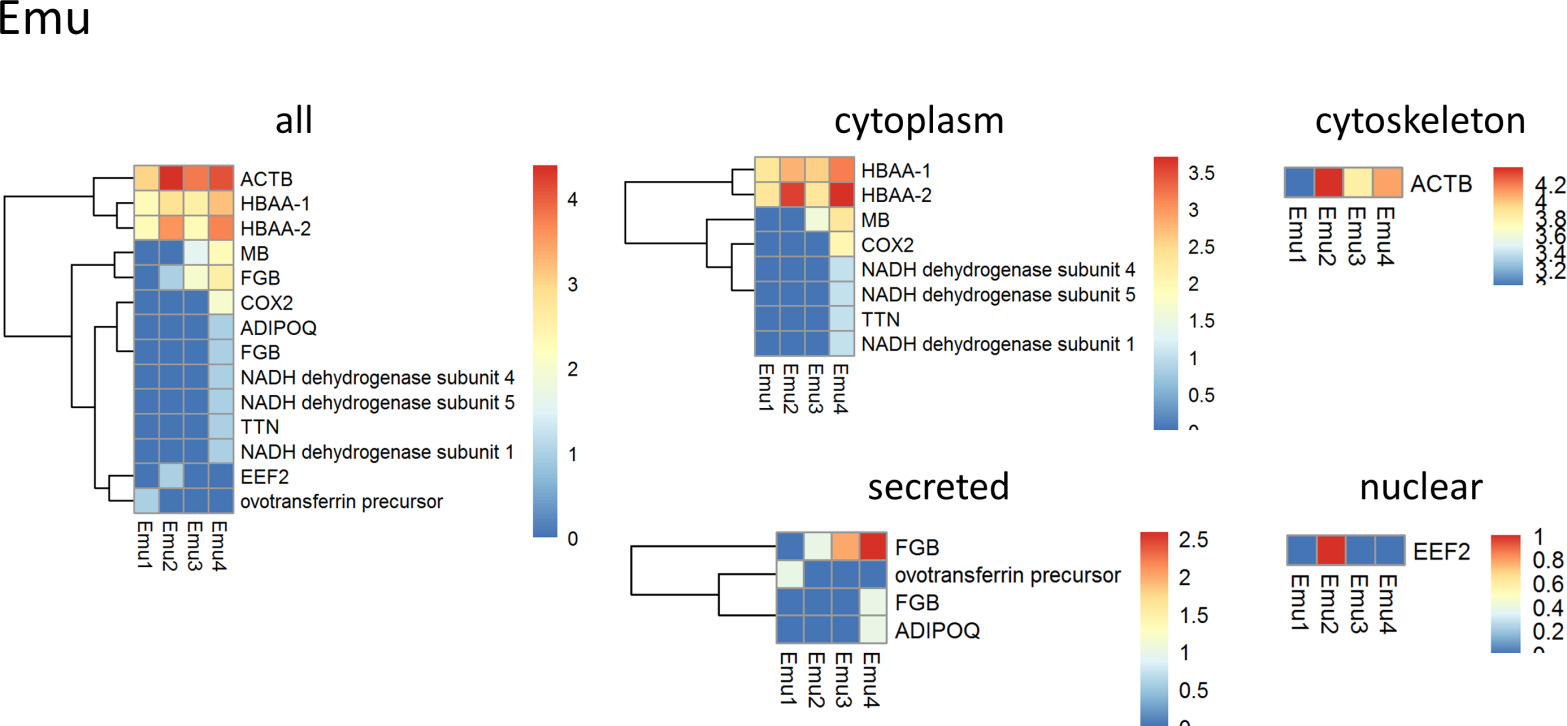
Mass spectrometric assessment of residual proteins following decellularization of emu lungs demonstrates overall concordance in residual proteins detected. Positively identified proteins in decellularized emu lungs (i.e. those proteins which were detected with at least 2 unique peptide hits and exceeded the FDR cutoff for identification) were assigned to groups according to subcellular location (cytoskeletal, cytosolic, ECM, membrane-associated, nuclear, secreted, and uncharacterized in case no subcellular location was specified). Heatmaps were generated using the log2 transformation of total peptide counts for all positively identified proteins and grouped by category. Representative heatmaps from 4 emu lungs are shown.

**Fig 9:**
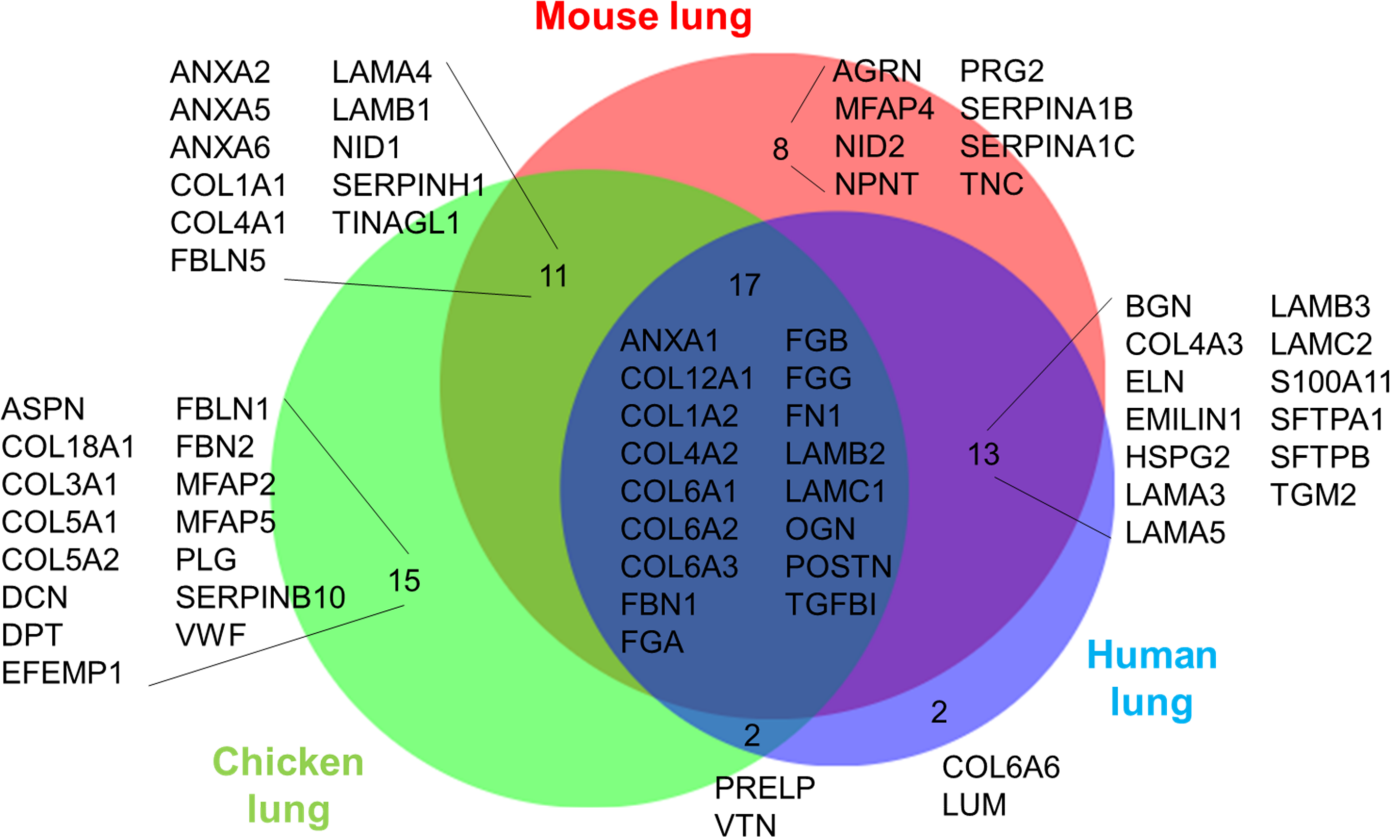
Matrisome comparison between species. Comparison of the matrisome proteins detected in different decellualrized lung tissues showed overlap of the most abundant lung proteins like collagen I, and IV, fibronectin, fibrillin, and laminins. Venn’s diagram shows the overlap of proteins allocated to the matrisome in MS datasets from decellularized lungs of mice, humans, and chickens.

Proteins positively identified with two or more unique peptide hits by mass spectrometric analyses of decellularized chicken and emu lungs were subsequently categorized into one of six groups based on cellular or extracellular location: cytosolic, ECM, cytoskeletal, nuclear, membrane-associated, or secreted [5, 7–10, 12]. In rare cases no classification could be assigned and therefore proteins were grouped as “uncharacterized”. If any of the proteins were matched to more than one category, we chose its predominant subcellular location for functional grouping. Heatmaps generated from the log2 transformation of unique peptide hits from each positively identified protein are depicted for visual comparison in Fig 7 and Fig 8. The number of total proteins identified in chicken (Fig 7) and emu (Fig 8) lungs is limited by available databases, particularly for emu lungs. As such, a total of 307 proteins were detected in decellularized chicken lungs with a variation of about 25% between individual lungs and detection of 185 ± 46 proteins/lung on average. Only 14 proteins were identified in decellularized emu lungs with a variation of about 37% between individual emu lungs and detection of 9 ± 3 proteins/lung on average. Individual peptide counts are depicted in S1 and S2 Tables. Notably, no ECM proteins were identified in decellularized emu lungs, again reflecting limitations in available databases. For the comparison of the similarities between the proteins of decellularized lung tissue in different species we focused on proteins allocated to the matrisome [39]. We performed the post analysis of the proteins that would be retained by the decellularization method in avian lungs comparing with murine and human dataset from former publications of our group [28,29]. Fig 9 shows that there is a major overlap in matrisome associated proteins detected in avian and mammalian lungs. Most abundant lung proteins such as collagen I, and IV, fibronectin, fibrillin, and multiple laminins were identified in all three species after decellularization.

### Human lung cells survive for varying times following inoculation into decellularized chicken vs emu lungs

**Fig 10:**
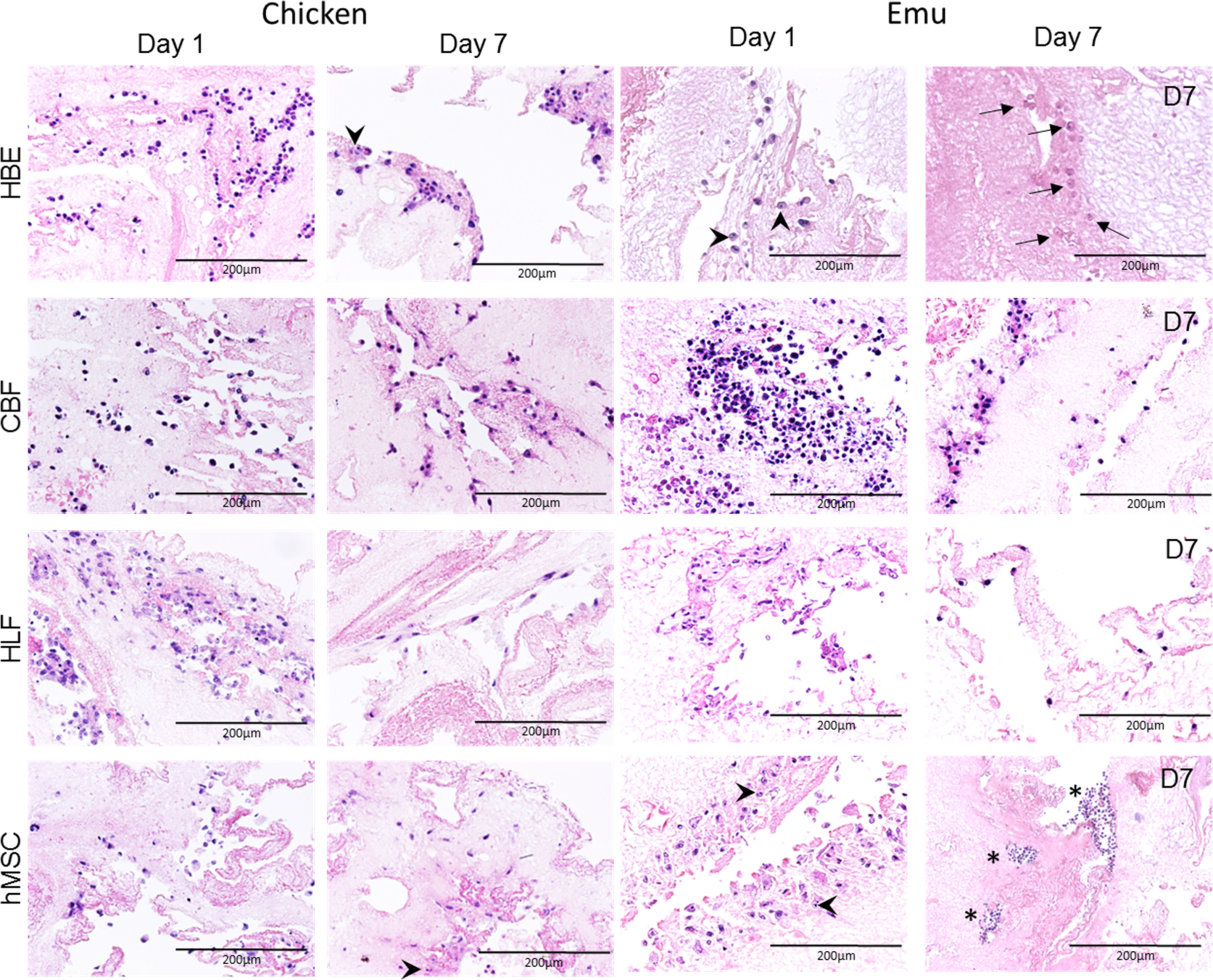
HBEs, hMSCs, CBFs, and HLFs demonstrate comparable initial seeding patterns but different growth patterns following inoculation into decellularized chicken and emu lungs. Representative H&E low power (100X) photomicrographs show characteristic recellularization patterns one day post-inoculation of each cell type in acellular chicken or emu lungs. Representative images from 3 chicken lungs and 3 emu lung segments seeded with each cell type are shown. In general, cells that do not interact with the ECM scaffold and remain in the airspaces or vascular spaces unattached to any matrix demonstrated rounding up of cells and nuclear fragmentation. Arrowheads indicate fragmented nuclei in cells that appear to be undergoing apoptosis. Arrows indicate cells that have no clear blue nuclear staining and thus appear not to be alive. Stars indicate locations of rounded and detached cells. Original magnification 200X, scale bars are indicated on each image.

**Fig 11:**
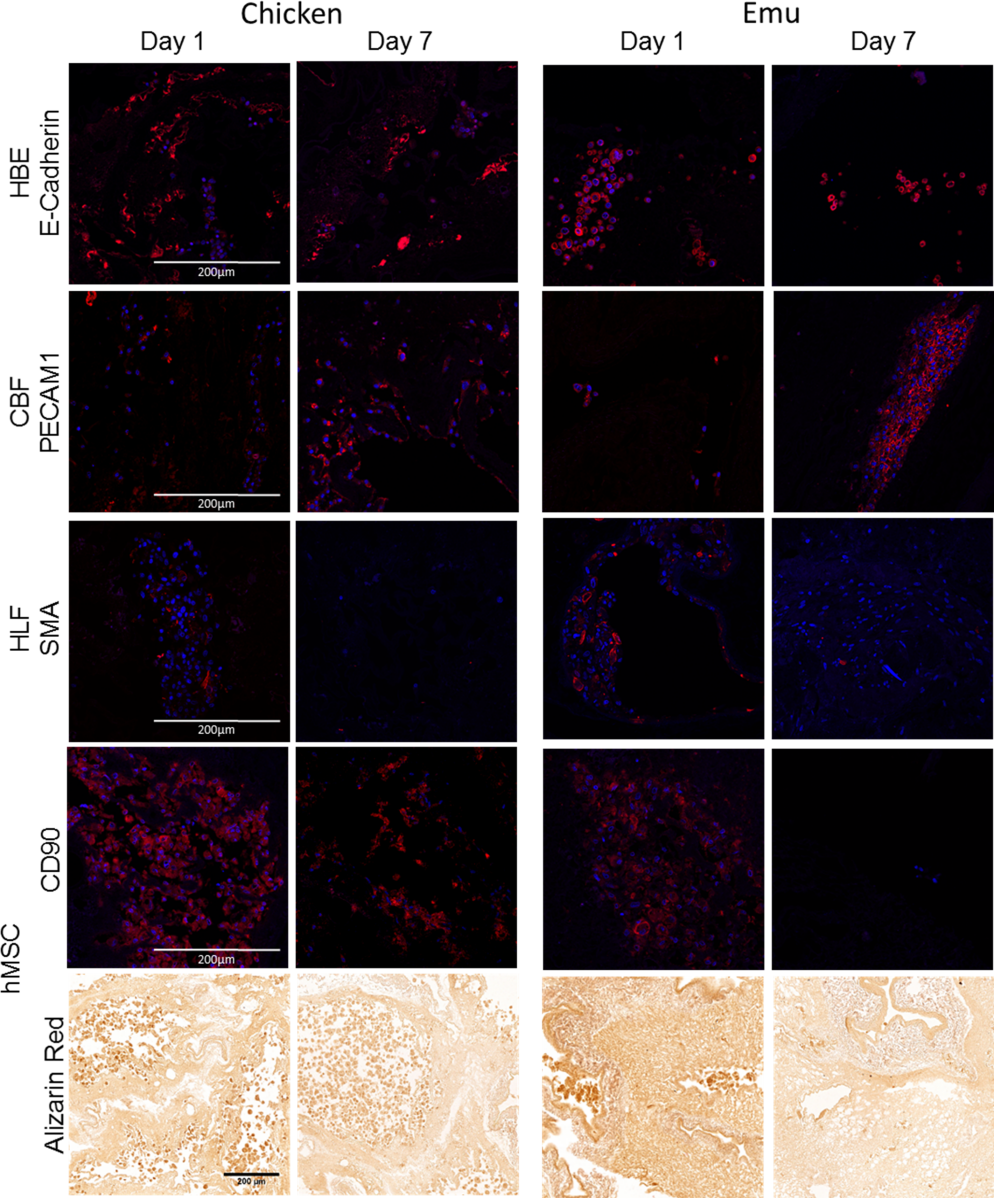
HBEs, CBFs, HLFs and hMSCs demonstrated cell phenotype maintenance in recellularized chicken and emu lungs. Representative fluorescence microscopy images of specific cell morphology biomarker for HBEs, CBFs, HLFs and hMSCs at day 1 and day 7. Stain of interest is depicted in red. Bright field images of repopulated lungs with hMSC did not show calcium deposition (commonly for osteogenic in hMSC differentiation) in dark-red (Alizarin red).

Using recellularization techniques previously described for both small and large mammalian models, inoculation of decellularized chicken and emu lungs was performed via a major airway or vascular conduit to either the entire organ (chicken) or 2–3 cm^3^ segments (emu) [5, 7, 8]. Initial cell binding, cellular localization and adhesion to the extracellular matrix, and cellular growth of the human cell lines were assessed histologically. Representative images of days 1 and 7 are displayed in Fig 10. All cells initially adhered to decellularized chicken scaffolds on day 1 and were found through day 7. In contrast, initial seeding (day 1) of both HBEs and hMSCs demonstrated weak attachment on the emu scaffolds and many cells appeared to be undergoing apoptotic changes. HBE cells were nearly absent of nuclear staining on day 7 on the emu lungs (Fig 10) while the stain was prevalent on the chicken lungs. In comparison to day 1, hMSC cells were rounded up and small on day 7. On both scaffolds, these cells were both viable up to day 7. Thereby, viable cells seeded on decellularized emu lungs were generally lower than on the chicken lungs. On the other hand, CBFs and HLFs demonstrated robust initial attachment to the emu scaffolds on day 1 and viability up to day 7. Fig 11 shows immunofluorescence images of different biomarkers positive for specific mature cell phenotype in recellularized chicken and emu lungs. In chickens, HBEs demonstrated formation of adherent junctions (E-cadherin^+^) at day 1 and day 7. CBFs revealed cell intercellular junctions commonly observed in endothelial cells (PECAM1^+^) but only at day 7, a weak signal was observed at day 1. And HLFs, marked positive for contractile proteins, as alpha smooth muscle actin (SMA^+^), were observed at day 1 and not at day 7. Cells seeded into emu lungs showed same behavior as in chicken with the exception of increased PECAM1 signal at day 7. HMSCs positive to CD90 (Thy-1) marker showed their multipotency in chickens at day 1 and day 7 and in emu at day 1. In addition to their multipotency state, no differentiation to osteoblast or calcium deposition was observed in cell regions for chicken and emu lungs by Alizarin red staining. However, a dark red (positive for calcium) was observed at the edges of the sections without any correlation with near or close-by hMSCS. This likely reflected reaction of the alizarin with the calcium alginate used as an artificial pleural coating.

### Assessment of proliferation (Ki67-staining) and apoptosis (Caspase 3-staining) of the cells after initial attachment on day 1 and after 7 days of incubation show cells proliferating on chicken scaffolds and apoptosing on emu tissue

**Fig 12:**
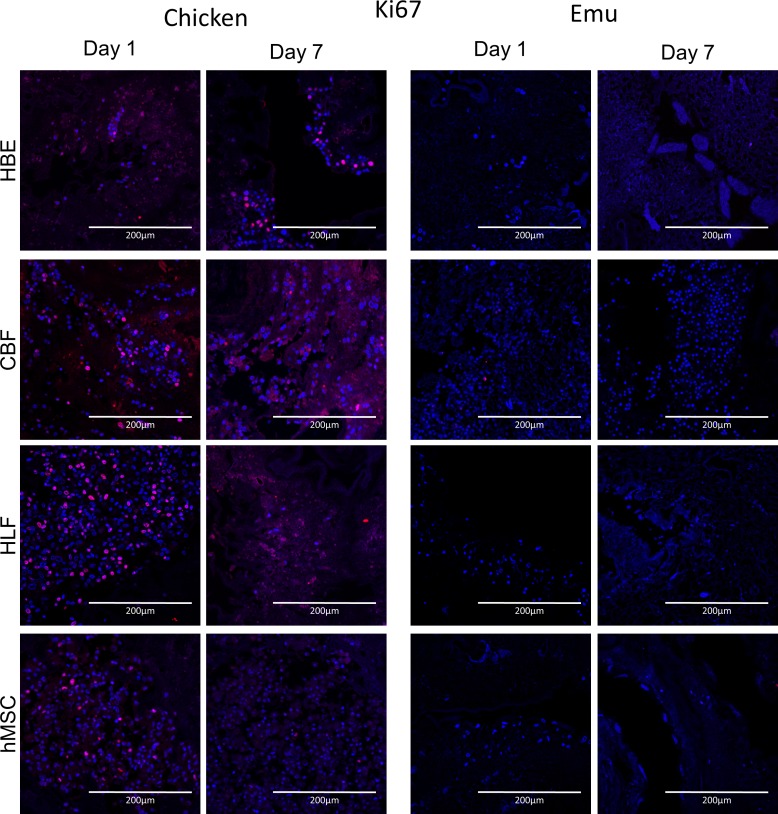
Cells seeded into decellularized chicken or emu lungs demonstrate similar patterns of Ki67. Representative photomicrographs of Ki67 staining day 1 and day 7 post-inoculation of each cell type. Ki67 staining is indicated in red and DAPI nuclear staining in blue. Representative images from 3 decellularized chicken and emu lungs seeded with each cell type are depicted. Original magnification 200X, scale bar 200 μm.

**Fig 13:**
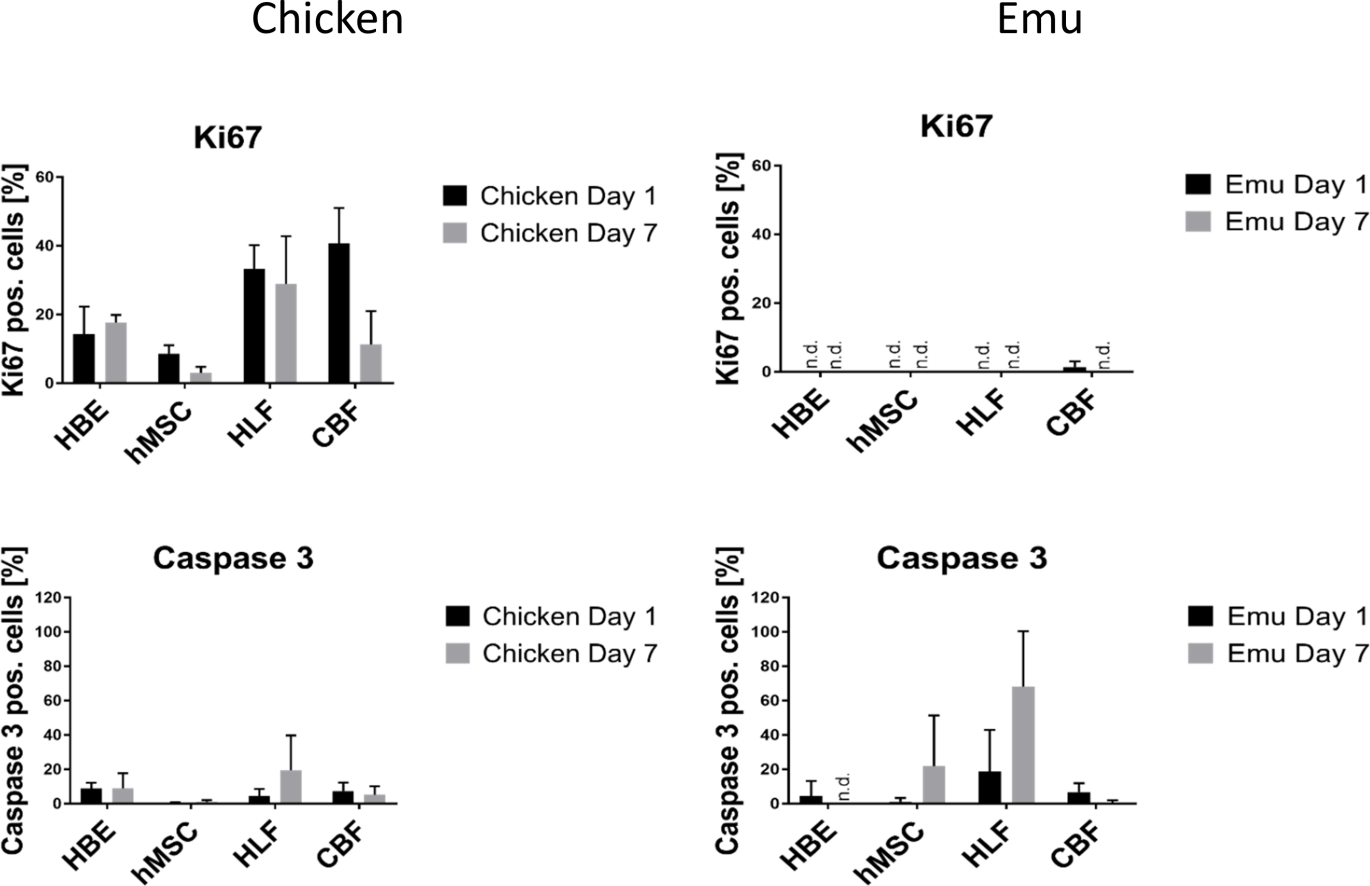
Quantitative analysis of cells seeded into decellularized chicken or emu lungs. Quantitative analysis of randomized images from 2 decellularized chicken lungs and 1 emu lung segment seeded with each individual cell type. 4 regions/slide from each seeding and time point were quantified to determine the percentage of ratio of positive stained Ki67 or caspase-3 expressing cells (red staining = Ki67/caspase-3) to total cells (blue staining = DAPI). Mean ± standard deviations of the different quantified regions are depicted.

**Fig 14:**
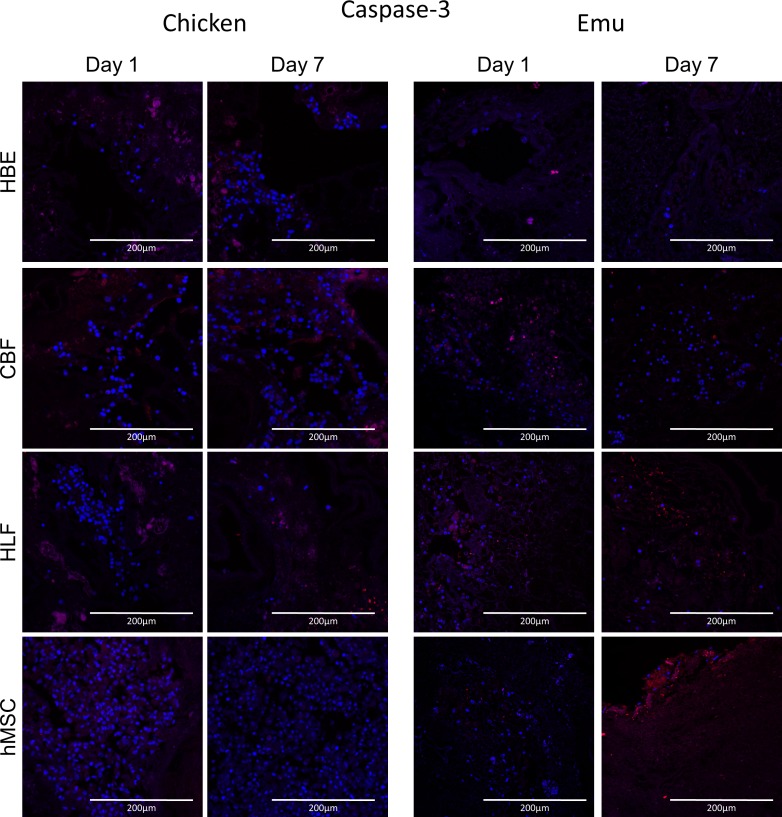
Cells seeded into decellularized chicken or emu lungs demonstrate similar patterns of caspase-3 staining. Representative photomicrographs of caspase-3 staining day 1 and day 7 post-inoculation of each cell type. Caspase-3 staining is indicated in red and DAPI nuclear staining in blue. Representative images from 3 decellularized chicken and emu lungs seeded with each cell type are depicted. Original magnification 200X, scale bar 200 μm.

Initial Ki-67 staining (day 1) (Fig 12) of the cells inoculated into the chicken scaffolds ranged between 10% and 40% with highest values for HLF and CBF cells (Fig 13). At day 7 no significant difference compared to day 1 was seen for all cell types although there was a trend to lower Ki67 staining especially for the CBF cells. Caspase 3 staining (Fig 14) was generally low for all cell types seeded onto the chicken scaffolds and was comparable between day 1 and day 7 (Fig 13). In contrast, low Ki67 staining was observed for any cell type seeded into the emu scaffolds on either day 1 or 7. However, significant amounts of caspase-3 staining were observed for all cell types on both day 1 and day 7. Further, there was a trend towards increased caspase-3 staining of HLFs and hMSCs on day 7 (Fig 14).

## Discussion

Significant progress has been made in the field of bioengineering using decellularized tissue from multiple mammalian species to generate tissues and organs for transplantation [38,40,41]. As such, decellularized lungs may become useful vectors for whole lung bioengineering and may help ameliorate a shortage of donor lungs needed for a growing population with severe lung disease and organ failure. However, much further work is needed to create a fully functional gas exchange unit based on decellularized mammalian lungs. As a potential alternative, the avian respiratory system, which is evolutionarily divergent from mammals with key differential structural and functional characteristics, has been argued to have superior gas exchange compared to mammalian respiratory systems [23–26]. Providing patients with a bioengineered organ for transplantation may therefore be achieved faster using avian lungs. As a foundation for further investigation into the use of decellularized avian lung scaffolds for potential xenotransplantation or other uses, we have successfully decellularized and characterized both small and large avian lungs and demonstrated initial recellularization with a range of human lung cell types.

The evolutionary divergence between avian and mammalian respiratory systems has led to unique mechanisms of avian ventilation and gas exchange. Driven by selection pressures and aerobic metabolism required for flight, the avian lung maximizes gas exchange and oxygenation in part through an extremely thin blood-gas barrier [42, 43]. Further, the avian respiratory system features a unidirectional airflow system in which inspired air is first directed to the posterior or caudal air sacs, followed by the lung itself, followed by the cranial or anterior air sacs, concluding with exhalation back through the trachea [44, 45]. While ectothermal crocodilian lizards also feature unidirectional airflow through their lungs, avian physiology has improved upon this to increase aerobic respiration and endothermal regulation [46]. Separating the ventilation component and gas exchange aspects into separate organs (the air sacs and lungs, respectively) permits unidirectional flow (via parabronchi, rather than alveoli), minimizing dead space ventilation, an inevitable consequence of tidal flow, protection from barotrauma [47], and presumably decreasing aspiration risk [24, 48]. While blood gas exchange units must be thin with a large surface area to permit adequate gas diffusion, both the transmural pressure from pulmonary capillaries and repeated distention and expansion of mammalian gas exchange units creates wall stress which may lead to mechanical failure [49]. The avian lung itself evolved a different solution to this issue—a relatively volume-constant, rigid, cross-current exchange mechanism similar to a radiator in design [49]. Although the physiological role in bird lungs is not well understood, surfactant is also produced by avian type II pneumocytes, regardless of the lack of distending end alveoli within the avian structure [46]. The limited changes observed in vascular airway resistances could be derived through the natural architecture of the avian lungs, which are not subjected to cyclic stretch (breathing air in and out) and described as static structures. This leads to the assumption of a rather constant resistance during the decellularization process.

Using a modification of the detergent-based techniques for decellularizing mammalian lungs, intact scaffolds were produced and assessed by a range of histologic, immunohistochemical, and mass spectrometric assessments. The presence of preserved collagen type I, collagen type IV, elastin, laminin, and fibronectin in the decellularized chicken lungs provides a similar framework to which a range of differentiated human lung epithelial, stromal, and pulmonary vascular endothelial cells or lung progenitor cells can adhere. In contrast, impairment of cellular attachment, proliferation, and survival was seen in the emu scaffolds. The scaffolds derived from emu lungs demonstrated similar retention of collagen in the Masson Trichrome staining as the chicken scaffolds, which was confirmed by immunohistochemical staining for collagen I and collagen IV. While laminin seemed to be preserved a reduction of elastin and fibronectin were observed specifically in the emu scaffolds. More detailed assessment of the remaining proteins in both the chicken and particularly the emu lungs by mass spectrometry were limited by the available databases. However, based on previous reported data [28,29] we compared the ECM matrisome between different species and our acellular chicken lungs. Despite the differences, an analysis of the extracellular matrix protein composition shows remarkable similarities of chicken ECM proteins compared to decellularized mammalian (rodent, non-human primate, pig, and human) tissue [5, 7, 10, 11, 13]. Due to limited size of the available emu database, a comparison was inadequate but similarities are expected. Notably, glycosaminoglycans were almost absent after the decellularization shown by Alcian Blue staining in both chicken and emu lungs. More detailed assessments of the remaining proteins and glycoproteins is needed, particularly to potentially help explain the discrepancy in cell survival and proliferation in decellularized chicken vs emu lungs. An adaptation of the decellularization agents (i.e. using different detergents) specifically to the emu tissue should further be considered to preserve more of the extracellular proteins that will be relevant for recellularization. Airway epithelial (HBE) and hMSCs, while viable on chicken scaffolds, were apoptotic on the emu tissue prior to day 7. In contrast, human lung fibroblasts (HLFs) and pulmonary vascular endothelial cells (CBFs) were viable for 7 days in both decellularized chicken and emu lungs but grew more in chicken lung scaffolds. HBEs and hMSC significantly rely on fibronectin for cellular attachment, reduction of apoptosis, and induction of proliferation [50–52]. Importantly HBEs, CBFs and HLFS demonstrated mature phenotype preservation [31–33] along day 1 and day 7 and hMSCs demonstrated the maintenances of its multipotency at day 1 and day 7 without any differentiation during this period [34]. The calcium depositions observed at some edges of the tissue sections marked by Alizarin red was an artifact likely derived from the crosslink solution (3% calcium chloride) used as artificial pleura [14]. The limited amount of cells observed could be an effect on survival/viability of the different cells due to the limitation of perfusion even when the tissue is assumed as porous and permeable with the possibilities to equilibrate to the incubator atmosphere characteristics of CO_2_ and O_2_ concentrations [14]. Moreover, the reduced amount of fibronectin in the emu scaffolds may be an explanation for the limited viability and proliferation of the HBE and hMSC on those scaffolds.

Further investigation of different cell types and their capability of recellularizing different decellularized avian scaffolds, in combination with the evaluation of the role of glycosaminoglycans, proteoglycans, and matrikines remaining in decellularized avian lung scaffolds, will be necessary to generate strategies for optimal repopulation with human cells. These approaches will include bioreactor culture systems, ECM derived hydrogels, and co-culture of different cell types.

In addition to these challenges in recellularization, further considerations are necessary in order to establish decellularized avian lungs as a potential biomaterial for human utilization. Primarily, the human tidal mechanism of diaphragm-based, bidirectional ventilation would need to be augmented to appropriate a unidirectional avian xenograft. Further, the air-sac system utilized by birds is not practical as a mechanism of ventilation in humans. As such, decellularized avian lungs may best serve as extracorporeal gas exchange devices, comparable to extracorporeal membrane oxygenation (ECMO) or dialysis [41–43]. Birds, similar to humans, possess natural antibodies to Galα1-3Galβ1-4GlcNAc (α-gal) epitopes, the major glycoprotein implicated in hyperacute rejection in mammal-to-human xenotransplantation [13]. The human immune response to the decellularized avian scaffold has not yet been studied. However, bird lungs do not express α-galactosylated proteins human serum demonstrates IgE to beef, pork, lamb, cat and dog but not to non-mammalian meat sources such as chicken, turkey or fish [53,54]. However, to consider decellularized avian lungs as a viable biomaterial for clinical use, further inquiry into the potential immunogenicity of the decellularized scaffolds is required [13]. Nonetheless, despite these questions, decellularized avian lungs offer a unique and innovative approach to *ex vivo* lung bioengineering.

## Conclusions

The revolution in whole organ tissue engineering has created novel techniques that make possible the use of decellularized xenogeneic tissue scaffolds for potential recellularization and transplantation [5]. While there remain significant hurdles to utilize these approaches for clinical lung transplantation, these techniques open the possibility of creating unique and functional biomaterials which can serve a broad array of clinical functions. While these models have traditionally been restricted to the mammalian class, the avian lung’s structural and physiological superiority to mammalian lungs represents a unique opportunity for synthetic bio-mimicry that may allow translation of the superior aerobic potential of the avian lung to human tissue oxygenation devices.

## Supporting information

S1 Fig Silicone injection molds reveal the chicken anatomy. Two silicone injection molds of chicken airways and airs sacs were cast. The first mold with air sacs attached is shown dorsally (I) and ventrally (II). The second mold without air sacs is shown from the same views (III and IV respectively), with a highly magnified ventral view of the parabronchi and parabronchia gas exchange microstructures (atria) (V).
(TIF)

S2 Fig DNA gels demonstrate minimal residual DNA in decellularized chicken and emu bird lungs compared to native controls. A) DNA ladder (M) and salmon sperm DNA (ssD, positive control) are shown for comparison. Nat = native, Representative gel and also quantitation of the DNA content in respective representative native and decellularized chicken (6) and emu (4) bird lungs are shown. B) Nuclear DAPI staining is depicted in blue for native tissue and non-visible nuclei were observed in decellularized tissue.
(TIF)

S3 Fig Semi-quantification of immunohistological staining. The fluorescence intensity of collagen I, collagen IV and elastin normalized to control values in (A) chicken and (B) emu, respectively. Values are presented as mean ± SD.
(TIF)

S4 Fig Controls for immunohistological staining. (A) No primary antibody control on native chicken and emu lung tissue for the respective antibodies indicated above each image. (B) Antibody positive controls with and without primary antibody using human gall bladder, kidney, liver, small bowel, and tonsil tissue. Collagen I, IV, laminin, fibronectin, elastin, Ki67, caspase 3, E-Cadherin, PECAM1, SMA and CD90 = red, DAPI = blue. Original magnification: 200x, scale bar: 100 µm.
(TIF)

S5 Fig Minimal residual anionic detergent (SDC) is detected in effluents from either chicken or elungs at the conclusion of the decellularization protocol. SDC concentration was calculated using a SDC standard curve, n = 7 for chicken (A) and n = 1 for emu (B).
(TIF)

S1 Table Total peptide counts for positively identified proteins in individual chicken bird lung samples, C = chicken. (PDF)

S2 Table Total peptide counts for positively identified proteins in individual emu bird lung samples. (PDF)
