# Supplementary figures and images for: Avian lungs: A novel scaffold for lung bioengineering
Source: PLoS One. 2018 Jun 27;13(6):e0198956. doi: 10.1371/journal.pone.0198956 (PMC6021073; doi:10.1371/journal.pone.0198956)

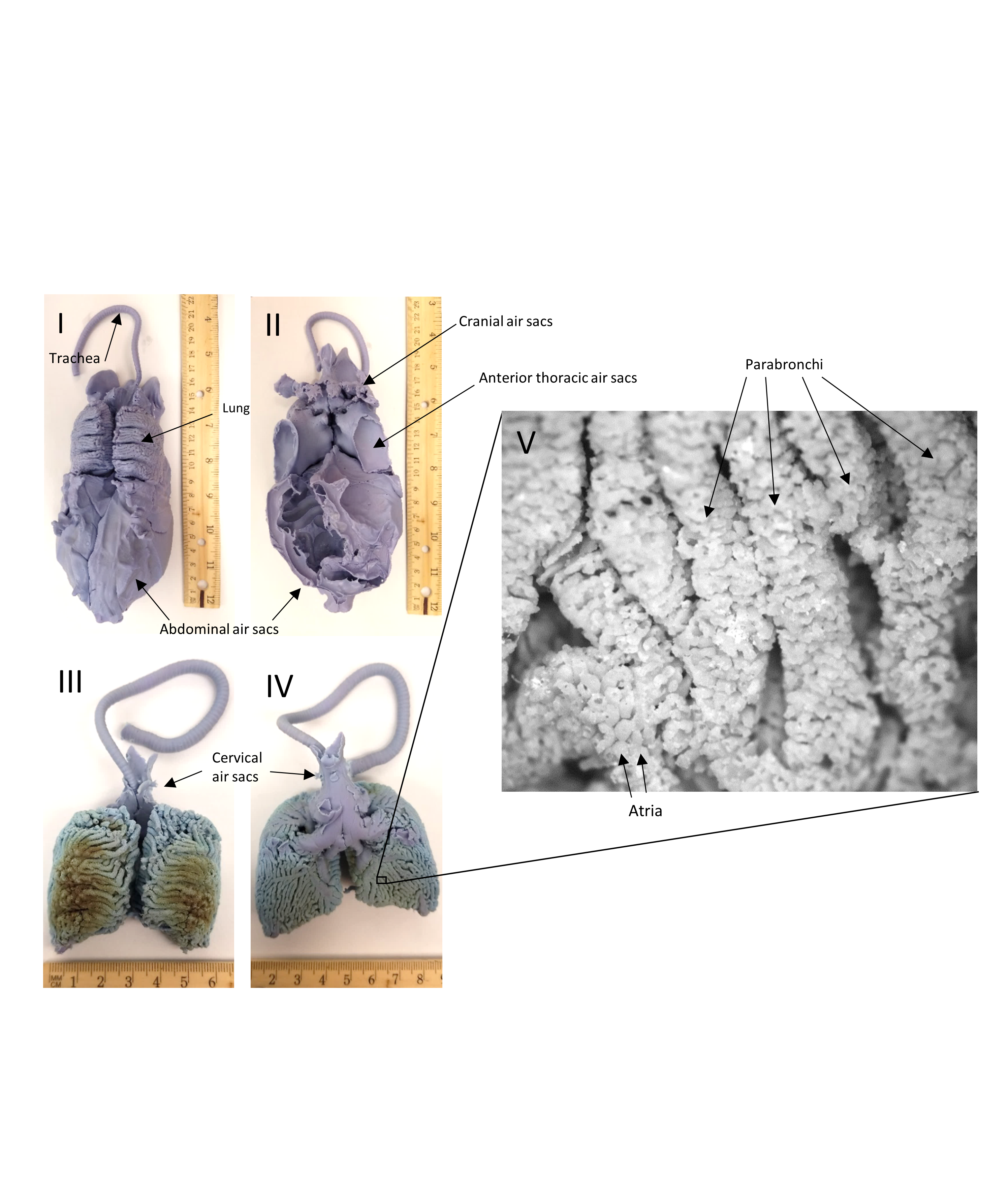

Supplement: S1 Fig — Two silicone injection molds of chicken airways and airs sacs were cast. The first mold with air sacs attached is shown dorsally (I) and ventrally (II). The second mold without air sacs is shown from the same views (III and IV respectively), with a highly magnified ventral view of the parabronchi and parabronchia gas exchange microstructures (atria) (V). (TIF) [file pone.0198956.s001.TIF]

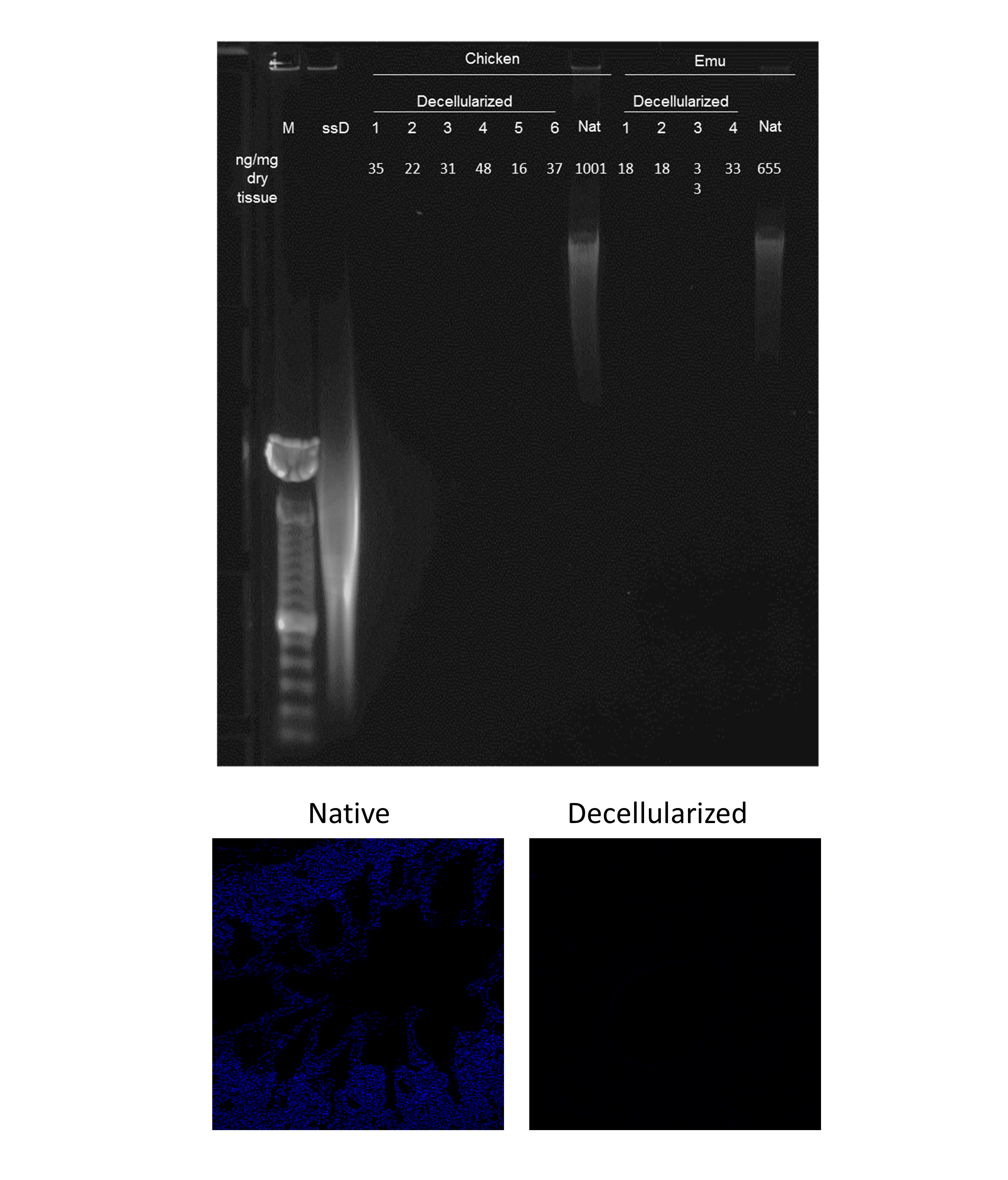

Supplement: S2 Fig — A) DNA ladder (M) and salmon sperm DNA (ssD, positive control) are shown for comparison. Nat = native, Representative gel and also quantitation of the DNA content in respective representative native and decellularized chicken (6) and emu (4) bird lungs are shown. B) Nuclear DAPI staining is depicted in blue for native tissue and non-visible nuclei were observed in decellularized tissue. (TIF) [file pone.0198956.s002.TIF]

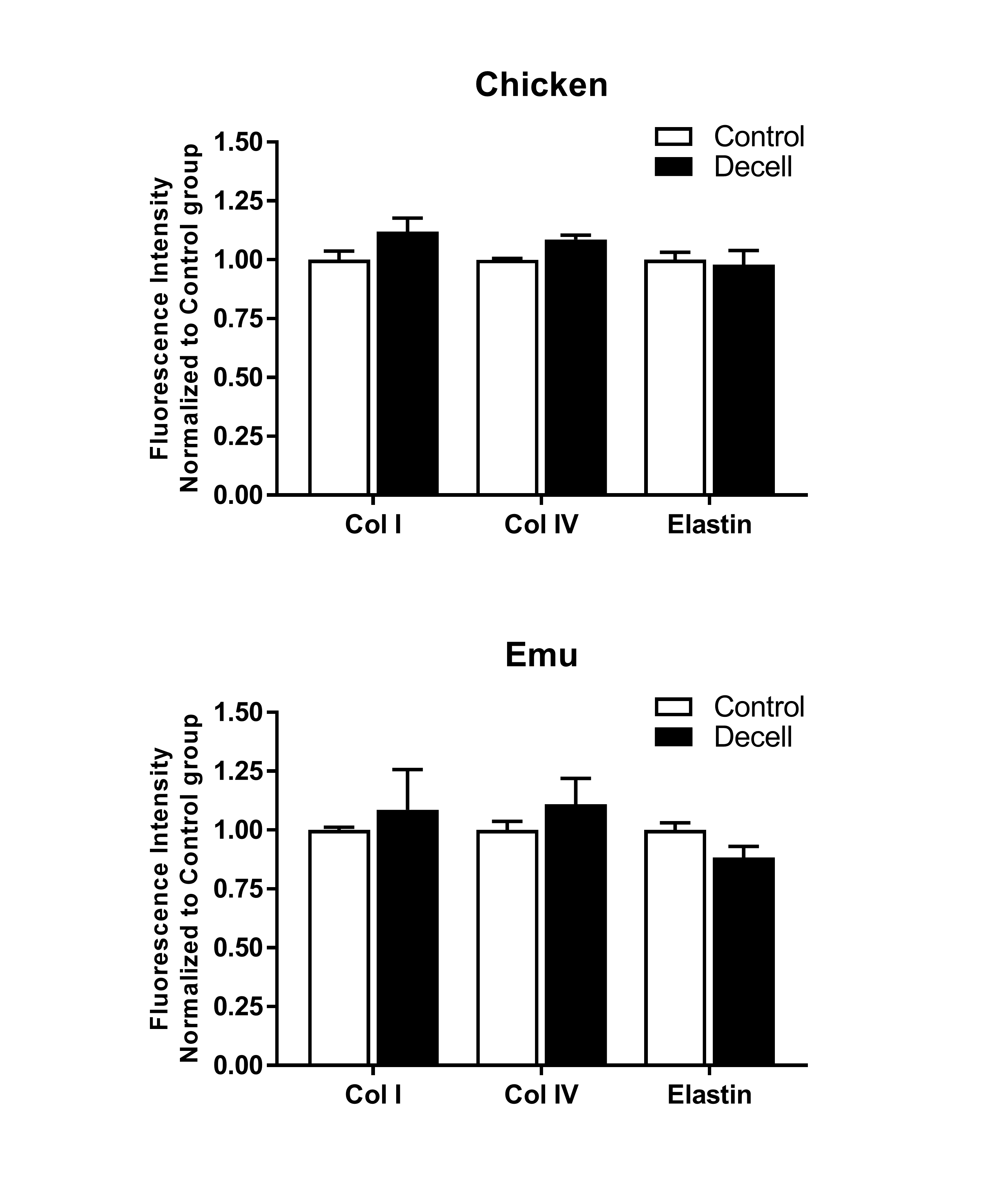

Supplement: S3 Fig — The fluorescence intensity of collagen I, collagen IV and elastin normalized to control values in (A) chicken and (B) emu, respectively. Values are presented as mean ± SD. (TIF) [file pone.0198956.s003.TIF]

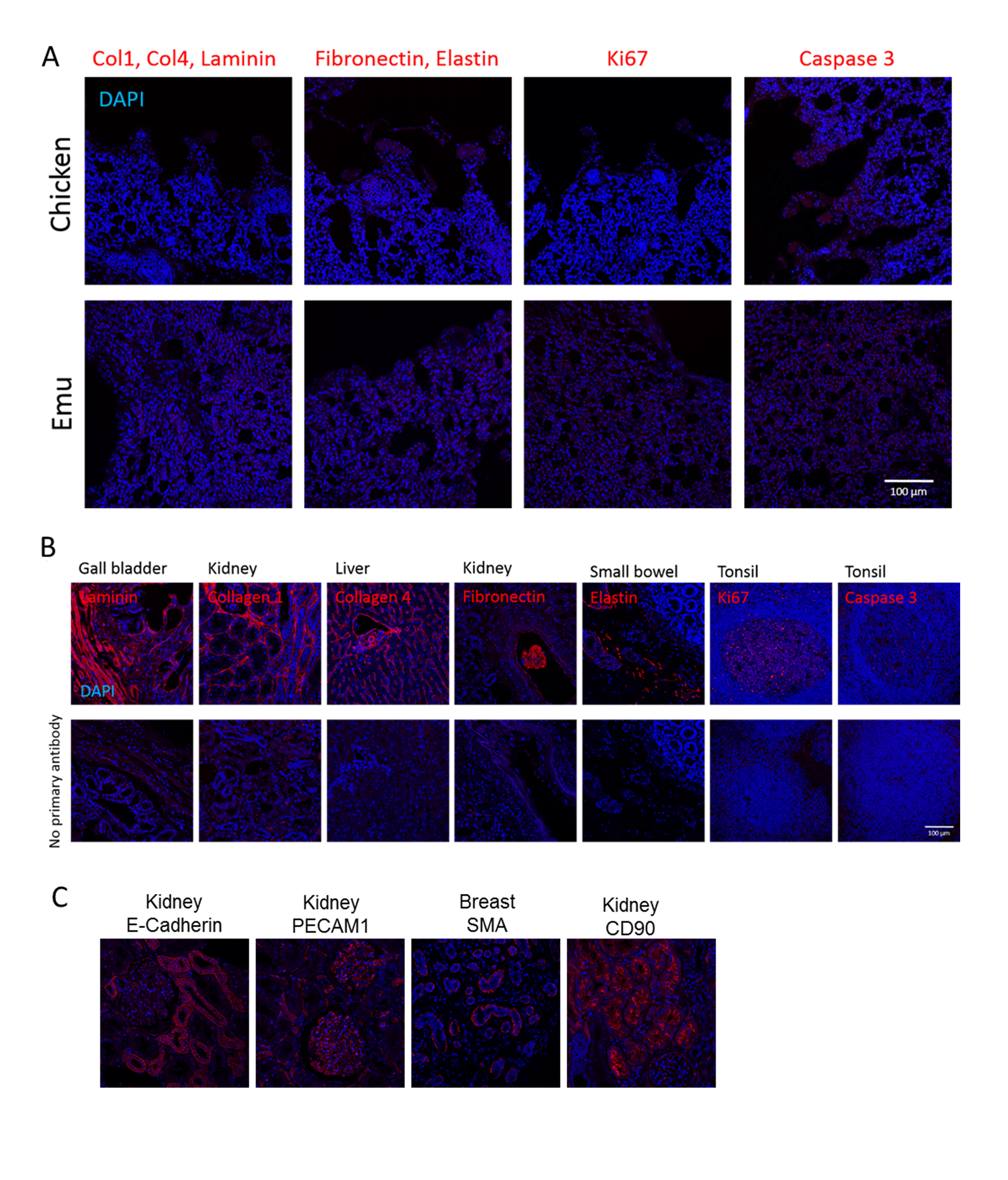

Supplement: S4 Fig — (A) No primary antibody control on native chicken and emu lung tissue for the respective antibodies indicated above each image. (B) Antibody positive controls with and without primary antibody using human gall bladder, kidney, liver, small bowel, and tonsil tissue. Collagen I, IV, laminin, fibronectin, elastin, Ki67, caspase 3, E-Cadherin, PECAM1, SMA and CD90 = red, DAPI = blue. Original magnification: 200x, scale bar: 100 µm. (TIF) [file pone.0198956.s004.TIF]

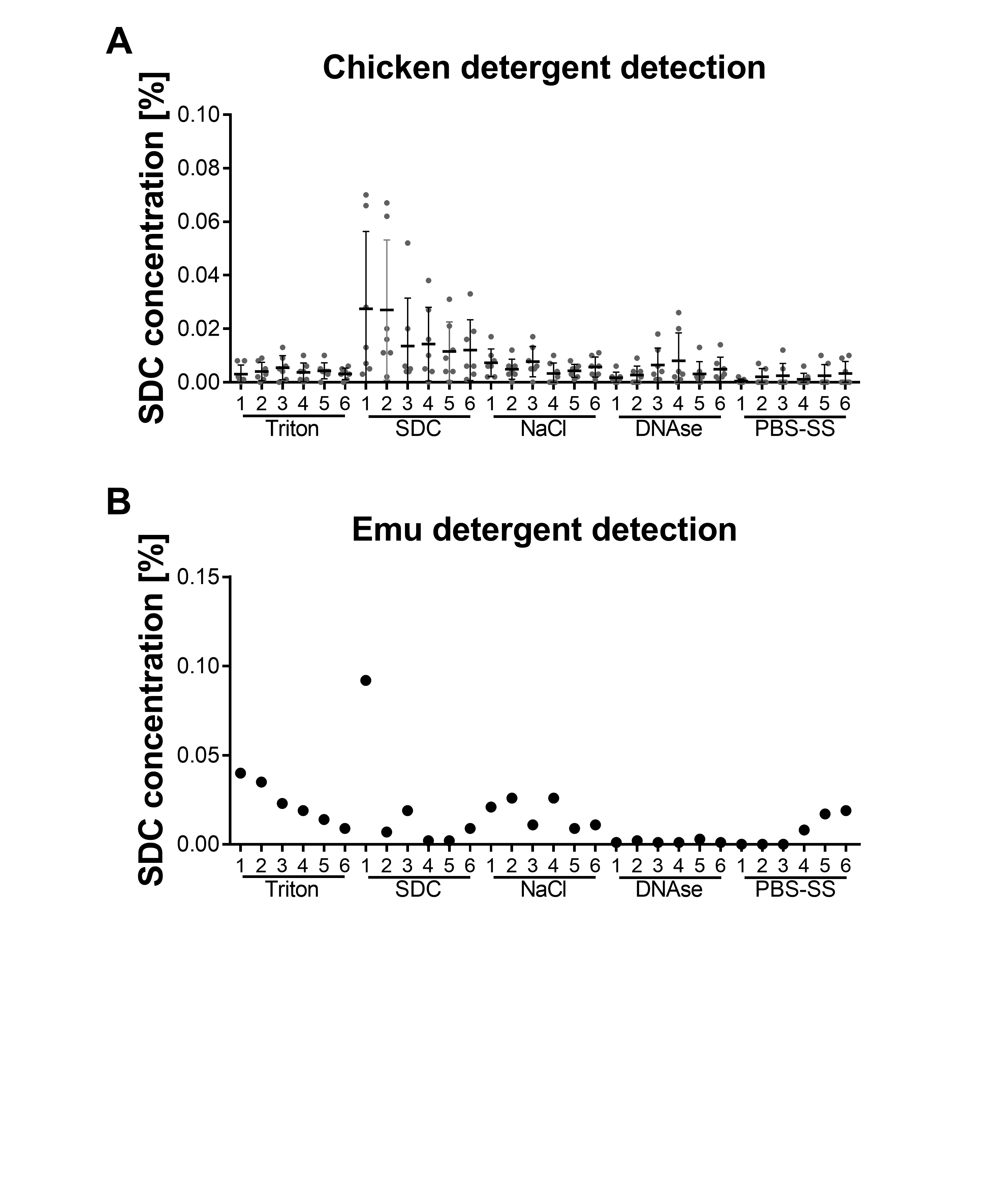

Supplement: S5 Fig — SDC concentration was calculated using a SDC standard curve, n = 7 for chicken (A) and n = 1 for emu (B). (TIF) [file pone.0198956.s005.TIF]
