# Supplementary material for: Avian lungs: A novel scaffold for lung bioengineering
Source: PLoS One. 2018 Jun 27;13(6):e0198956. doi: 10.1371/journal.pone.0198956 (PMC6021073; doi:10.1371/journal.pone.0198956)
Supplement: S1 Table — (PDF) [file pone.0198956.s006.pdf]

S1 Table: Total peptide counts for positively identified proteins in individual chicken lung samples, C=chicken.

Accession Number	Protein description	Subcellular location	Gene symbol	Molecular weight [kDa]	Σ# Unique Peptides	C1	C2	C3	C4	C5	C6
F1P4N9	Uncharacterized protein	cytoplasm	POSTN	94.1	2	16	22				17
F1N8W3	Uncharacterized protein	cytoplasm	POSTN	96.0	2	16	22	12	21	24	17
E1BW78	Uncharacterized protein	cytoplasm	COX6C	8.8	2	2	1		1	1	
E1C3B2	Cytochrome c oxidase subunit 6A, mitochondrial	cytoplasm	COX6A1	11.8	2	2	1				
F1NIL3	Uncharacterized protein (Fragment)	cytoplasm	ATP5L	11.7	2	1			1		
F1N9W9	Uncharacterized protein (Fragment)	cytoplasm	RPS11	18.2	2		1			2	
R4GIS7	Uncharacterized protein	cytoplasm	NDUFS2	14.1	2	1	1				
F1NU56	Uncharacterized protein	cytoplasm	RPS25	13.7	2	2				1	
H9KYU8	Uncharacterized protein	cytoplasm	not defined	21.4	2		1			1	1
R4GGJ0	Uncharacterized protein	cytoplasm	RPS16	16.4	2	2	1				
P08106	Heat shock 70 kDa protein	cytoplasm	not defined	69.7	2	3	4	7	6	2	2
E1C658	Uncharacterized protein (Fragment)	cytoplasm	ATP5H	18.4	2		2				
P05081	Adenylate kinase isoenzyme 1	cytoplasm	AK1	21.7	2			2	1		
P51417	60S ribosomal protein L15 (Fragment)	cytoplasm	RPL15	19.3	2	1					1
P26990	ADP-ribosylation factor 6	cytoplasm	ARF6	20.1	2		1		1	1	
E1C6C9	Uncharacterized protein	cytoplasm	NDUFB10	20.5	2	2	2		1		
P49702	ADP-ribosylation factor 5	cytoplasm	ARF5	20.5	2	1			2		
Q5ZMD1	14-3-3 protein theta	cytoplasm	YWHAQ	27.8	2	1				1	
E1BR10	Uncharacterized protein	cytoplasm	PRDX3	28.0	2	2			1	2	1
F2Z4L5	60S ribosomal protein L7a (Fragment)	cytoplasm	RPL7A	29.8	2	2	1				
Q5F3B9	Succinyl-CoA ligase subunit beta	cytoplasm	SUCLA2	50.0	2	1		1			
E1BUZ0	Uncharacterized protein	cytoplasm	NDUFV2	27.1	2	1	2				
F1N9U8	Uncharacterized protein	cytoplasm	ETFA	36.6	2		1	1			

E1C2E3	Uncharacterized protein (Fragment)	cytoplasm	VDAC3	30.5	2	2	1		2	1	
F2Z4K7	40S ribosomal protein S3a	cytoplasm	RPS3A	29.8	2	1				1	
F1NJI0	Annexin (Fragment)	cytoplasm	ANXA5	36.2	2	1			1		
F1P3F9	Glutamate dehydrogenase	cytoplasm	GLUD1	47.6	2	1		2	1		
Q5ZL56	Uncharacterized protein	cytoplasm	ACADS	44.6	2			1	1		
H9KZ88	Guanine nucleotide-binding protein G(i) subunit alpha-2 (Fragment)	cytoplasm	GNAI2	39.0	2	2			1		
Q5ZLR5	Cytochrome b-c1 complex subunit Rieske, mitochondrial	cytoplasm	UQCRFS1	29.4	2	1	1		1		
R4GM10	Fructose-bisphosphate aldolase	cytoplasm	ALDOC	39.3	2			2			
P18940	NADH-ubiquinone oxidoreductase chain 5	cytoplasm	MT-ND5	66.3	2	1	1	1	1		
Q5F426	Pyruvate dehydrogenase E1 component subunit alpha	cytoplasm	PDHA1	44.4	2	2			1		
F1NC38	Uncharacterized protein	cytoplasm	ACADL	47.8	2	2			1		
Q5ZL72	60 kDa heat shock protein, mitochondrial	cytoplasm	HSPD1	60.9	2	1			2		
E1C3Y5	Protein transport protein Sec23A	cytoplasm	SEC23A	86.1	2	2				1	1
F1P130	Uncharacterized protein (Fragment)	cytoplasm	Gga.11316	102.3	2				2		
Q5ZJA7	Uncharacterized protein	cytoplasm	OGDH	115.1	2	1			1		
E1BSA7	Uncharacterized protein	cytoplasm	SEC24A	120.0	2		1				1
F1NLS6	Focal adhesion kinase 1	cytoplasm	PTK2	118.9	2		1			1	2
F1NSR9	Uncharacterized protein	cytoplasm	RYR3	552.3	2			2			
P29616	Myosin heavy chain, cardiac muscle isoform (Fragment)	cytoplasm	not defined	127.9	3	97					
P0CG62	Polyubiquitin-B	cytoplasm	UBB	34.3	3	3	3	1	2		
E1BT94	Uncharacterized protein	cytoplasm	NDUFB6	14.9	3	1	2				
P67883	60S ribosomal protein L30	cytoplasm	RPL30	12.8	3	2	2		2	3	2
F1NJ10	Uncharacterized protein (Fragment)	cytoplasm	SLC25A5	35.0	3	9	4				1
Q90835	Elongation factor 1-alpha 1	cytoplasm	EEF1A	50.1	3	6	6	5	7	7	4

R4GHG4	Succinate dehydrogenase [ubiquinone] iron-sulfur subunit, mitochondrial	cytoplasm	not defined	23.9	3	3	1				
F1NCI6	60S ribosomal protein L7 (Fragment)	cytoplasm	RPL7	28.7	3	2	1	1	1		
F1NWP3	Heat shock cognate 71 kDa protein	cytoplasm	HSPA8	70.8	3	3	4	7	5	2	2
Q5ZIT5	Ras-related protein Rab-10	cytoplasm	RAB10	22.5	3	2	1	1	1		
E1C043	Uncharacterized protein	cytoplasm	COX5A	17.0	3	3	1				
F1NZ78	Alpha-enolase	cytoplasm	ENO1	47.3	3	1		3	1		
P05122	Creatine kinase B-type	Cytoplasm	CKB	42.8	3		1		3		
F1NJ97	Isocitrate dehydrogenase [NAD] subunit, mitochondrial (Fragment)	cytoplasm	IDH3A	39.3	3	2	1		1		
E1BUH7	Uncharacterized protein	cytoplasm	TMEM43	44.8	3	2	2				
F1NHH9	D-beta-hydroxybutyrate dehydrogenase, mitochondrial	cytoplasm	BDH1	38.0	3	3	1			1	
R4GLP7	Citrate synthase	cytoplasm	not defined	42.5	3	3			1	1	
P38024	Multifunctional protein ADE2	cytoplasm	AIRC	47.2	3	2	2			1	
P13731	Serpin H1	cytoplasm	SERPINH1	45.7	3	2	3		1		1
E1BT93	Uncharacterized protein (Fragment)	cytoplasm	ALDH2	57.3	3	2			1		1
E1C155	Uncharacterized protein	cytoplasm	ALDH6A1	58.3	3	3	1				
Q8QH01	Dimethylaniline monooxygenase [N-oxide-forming]	cytoplasm	FMO3	60.4	3	2	1				
F1NWB7	Endoplasmin	cytoplasm	HSP90B1	91.5	3	3			1		
F1NVF0	Uncharacterized protein	cytoplasm	VPS35	91.7	3	1	2				
E1BUD8	Uncharacterized protein	cytoplasm	SEC24C	121.6	3	1	3		2		
F1P593	Heat shock protein beta-1	cytoplasm	HSPB1	21.8	4	2	2	2	2		
Q5ZJV5	Uncharacterized protein	cytoplasm	COX4I1	19.6	4	2	2				
P07322	Beta-enolase	cytoplasm	ENO3	47.2	4	1		5			
Q5ZI00	Uncharacterized protein	cytoplasm	NDUFA9	43.1	4	2	3				
Q5ZIQ3	Heterogeneous nuclear ribonucleoprotein K	cytoplasm	HNRNPK	47.2	4	4	1				
E1BTT8	L-lactate dehydrogenase	cytoplasm	LDHA	36.5	4			3	1		

F1P3U1	Uncharacterized protein	cytoplasm	IMMT	79.2	4	2	2		1		
F1P0N2	AMP deaminase 1	cytoplasm	AMPD1	85.7	4			4			
Q90635	Dihydropyrimidinase-related protein 2	cytoplasm	DPYSL2	62.3	4	3	1		2	2	
F1NW23	Uncharacterized protein (Fragment)	cytoplasm	CLTC	189.9	4	3	1		1		
F1ND23	Uncharacterized protein (Fragment)	cytoplasm	NDUFS3	29.2	5	3	2			1	
F1NED9	Uncharacterized protein (Fragment)	cytoplasm	FHL1	34.0	5	1	2	4			
Q5ZMH1	Septin-2	cytoplasm	SEPT2	40.2	5	2	5				
F1NQT9	Calsequestrin	cytoplasm	CASQ2	47.1	5	1	1	5			
H9L011	Uncharacterized protein	cytoplasm	LOC426023	47.5	5	3	2	1	3	1	1
Q5F420	Uncharacterized protein	cytoplasm	ACSL1	78.5	5	2	1	4	3		
P18944	Cytochrome c oxidase subunit 2	cytoplasm	MT-CO2	25.6	6	3	3	3	2	1	
P19966	Transgelin	cytoplasm	TAGLN	22.3	6	3	5	1	2	4	3
Q5ZLD1	Uncharacterized protein	cytoplasm	FH	54.3	6	2		2	4		
H9KZF8	ATP synthase subunit gamma	cytoplasm	ATP5C1	32.7	6	3		2	4	2	
F1NAC6	Uncharacterized protein	cytoplasm	UQCRC1	52.7	6	5	2		1		
F1N9Z7	Succinyl-CoA:3-ketoacid-coenzyme A transferase	cytoplasm	OXCT1	56.1	6	4	1		4		
F1NVG9	Annexin	cytoplasm	ANXA6	75.2	6	5	2	2	3		
F1P3D8	Uncharacterized protein	cytoplasm	SLC25A12	77.3	6	3		4	2		1
F1NXN8	Uncharacterized protein	cytoplasm	NDUFS1	79.5	6	6	1				
P11501	Heat shock protein HSP 90-alpha	cytoplasm	HSP90AA1	84.0	6	3			3	2	1
F1NZJ2	Hexokinase	cytoplasm	HK1	102.7	6	2		4			
Q5ZMJ6	Uncharacterized protein	cytoplasm	SLC25A4	32.9	7	12	5	6	11	7	1
P84172	Elongation factor Tu, mitochondrial (Fragment)	cytoplasm	TUFM	38.2	7	5	5	6	1		
E1BVT3	Malate dehydrogenase	cytoplasm	MDH2	35.6	7	6		3			
R9PXN1	Eukaryotic initiation factor 4A-II	cytoplasm	EIF4A2	43.5	7	6	1	1	3	2	
F1NZ24	Uncharacterized protein	cytoplasm	SLC25A3	40.0	7	4	1	2	2	1	

F1NPJ4	Succinate dehydrogenase [ubiquinone] flavoprotein subunit, mitochondrial	cytoplasm	SDHA	72.9	7	8	4		2		
F1NSC1	Uncharacterized protein	cytoplasm	ATP5F1	28.6	8	6	4		2		1
R4GF49	Uncharacterized protein (Fragment)	cytoplasm	NDUFV1	47.5	8	5	4				
F1NY29	Uncharacterized protein	cytoplasm	ETFDH	53.1	8	7	2				
Q90577	Sarcalumenin	cytoplasm	SRL	54.1	8	1		7	5		
P07630	Carbonic anhydrase 2	cytoplasm	CA2	29.0	9	8	4	1	3	1	
Q9I9D1	Uncharacterized protein	cytoplasm	VDAC2	30.2	9	10	9	5	7	6	5
F1NU17	Phosphoglycerate kinase	cytoplasm	PGK1	44.6	9	3		8	3		
F1P582	Uncharacterized protein	cytoplasm	UQCRC2	48.5	9	6	8	2	2	3	1
F1NFS0	Elongation factor 2 (Fragment)	cytoplasm	EEF2	95.2	9	8	2	3	2	1	2
P0CB50	Peroxiredoxin-1	cytoplasm	PRDX1	22.3	10	8	7	4	5	7	6
F1P180	Aspartate aminotransferase	cytoplasm	GOT2	47.3	10	8		3	5		
Q5ZMW1	Aconitate hydratase, mitochondrial	cytoplasm	ACO2	85.6	11	6		7	5	3	2
E1BSN7	Alpha-1,4 glucan phosphorylase	cytoplasm	PYGB	96.7	11	6	5	3	3		
P02112	Hemoglobin subunit beta	cytoplasm	HBB	16.5	12	10	10	8	11	8	8
E1BTT4	Uncharacterized protein	cytoplasm	HADHB	50.8	12	8	7	1	2	1	
F1NEF6	Uncharacterized protein (Fragment)	cytoplasm	ACAD9	67.8	12	8	2	3	9	1	1
F1NPA9	Uncharacterized protein	cytoplasm	RPS3	26.7	13	8	6	2	7	10	5
Q03669	Sarcoplasmic/endoplasmic reticulum calcium ATPase 2	cytoplasm	ATP2A2	114.6	13	21	14	19	15	11	2
E1C6A1	Uncharacterized protein	cytoplasm	NNT	113.7	13	5	8	9	6	3	1
F1NH87	Glyceraldehyde-3-phosphate dehydrogenase (Fragment)	cytoplasm	GAPDH	34.9	14	10	7	13	7	5	2
P13585	Sarcoplasmic/endoplasmic reticulum calcium ATPase 1	cytoplasm	ATP2A1	109.0	14		6	27			2
P00548	Pyruvate kinase PKM	cytoplasm	PKM	58.0	14	1		12	1	2	
F1NI29	Uncharacterized protein	cytoplasm	HADHA	85.7	14	11	8	1	7	4	
E1BT53	Uncharacterized protein	cytoplasm	Gga.51341	992.0	14			14			
F1NI22	ATP synthase subunit alpha	cytoplasm	ATP5A1	59.9	15	9	8	5	13	7	5

R4GL15	ATP-dependent 6-phosphofructokinase	cytoplasm	PFKM	85.1	15	3	2	15	2	1	
F1P1U6	Uncharacterized protein	cytoplasm	MPO	82.6	17	7	14		7	8	1
H9L340	ATP synthase subunit beta (Fragment)	cytoplasm	ATP5B	52.9	18	15	13	11	15	11	3
H9L366	Isocitrate dehydrogenase [NADP]	cytoplasm	IDH2	50.4	19	13	7	9	11	8	3
F1NJ08	Vimentin	cytoplasm	VIM	53.2	23	10	10	11	16	5	4
F1NJR5	Uncharacterized protein	cytoplasm	Gga.55808	212.5	23	2	13	4	17	3	3
P20111	Alpha-actinin-2	cytoplasm	ACTN2	104.2	32	40	26	33	22	14	5
P32882	Tubulin beta-2 chain	cytoskeleton	not defined	49.9	2	16	12		11		
R4GIG1	Uncharacterized protein	cytoskeleton	MYH1D	222.8	2		39	122	38		12
F1N9J7	Uncharacterized protein	cytoskeleton	LOC425049	50.0	2			7	7		5
F1NYU0	Uncharacterized protein	cytoskeleton	MYL2	18.7	2		3				
P09860	Troponin C, slow skeletal and cardiac muscles	cytoskeleton	TNNC1	18.4	2					2	1
R4GI85	Calponin (Fragment)	cytoskeleton	CNN2	31.0	2	2					
O93532	Keratin, type II cytoskeletal cochleal	cytoskeleton	not defined	53.8	2	4	3	1	4	3	1
Q5ZLC7	Microtubule-associated protein RP/EB family member 1	cytoskeleton	MAPRE1	29.1	2	2	1				
F1P4R3	Uncharacterized protein	cytoskeleton	FHL2	32.4	2	2					
H9L107	Uncharacterized protein (Fragment)	cytoskeleton	KRT4	58.7	2		2		3	3	
F1NRM5	Actin-related protein 2 (Fragment)	cytoskeleton	ACTR2	43.0	2	1		1	1	1	
R4GHW9	Erythroid protein 4.1	cytoskeleton	EPB41	96.8	2	2	1				
P11533	Dystrophin	cytoskeleton	DMD	422.6	2			2			
F1NKL4	Uncharacterized protein	cytoskeleton	DYNC1H1	525.5	2	2					
F1P476	Actin, gamma-enteric smooth muscle	cytoskeleton	ACTG2	42.0	3	18				11	5
P68139	Actin, alpha skeletal muscle	cytoskeleton	ACTA1	42.0	3	17	15	17			
F1N8P3	Uncharacterized protein	cytoskeleton	ACTBL2	42.0	3	16	14	12	12	8	
R4GJ21	Uncharacterized protein	cytoskeleton	MYH1C	223.2	3	38	54	152	39	22	
P09244	Tubulin beta-7 chain	cytoskeleton	not defined	49.6	3	18		10	13	10	9
F1NYB1	Uncharacterized protein	cytoskeleton	TUBB4B	49.8	3	17	12	11	11	10	8

F1P3W8	Uncharacterized protein	cytoskeleton	MYH1B	223.4	3			143			
P02552	Tubulin alpha-1 chain (Fragment)	cytoskeleton	not defined	45.9	3	13	10	9	9	7	7
A4UNW1	Myosin light chain 2	cytoskeleton	Myl2	18.8	3	1	4		3	1	
P02609	Myosin regulatory light chain 2, skeletal muscle isoform	cytoskeleton	MYLPF	18.8	3			3			
E1BSX2	Calponin	cytoskeleton	CNN3	28.4	3	3	2		1		
F1NK75	Uncharacterized protein	cytoskeleton	not defined	32.8	3	1	2				
F1NT51	Caldesmon	cytoskeleton	CALD1	86.4	3	1	3				
F1NG39	Unconventional myosin-Ic	cytoskeleton	MYO1C	118.8	3	3		1			
F1NJM8	Uncharacterized protein	cytoskeleton	SYNM	182.3	3			3			
R4GMK5	Uncharacterized protein	cytoskeleton	MYH1E	223.4	4	36	47	119	33	21	
E1BZ05	Desmin	cytoskeleton	DES	48.8	4	12	17	9	17	7	6
F1NDN5	Uncharacterized protein	cytoskeleton	KRT15	47.9	4	2		1	5	2	
Q04205	Tensin	cytoskeleton	TNS	187.1	4	1	3		1		1
F1N8D4	Uncharacterized protein	cytoskeleton	FLNB	277.0	4	3	3	2			
P26932	Calponin-1	cytoskeleton	CNN1	32.3	5	5	3		1		2
F1P5H0	Uncharacterized protein	cytoskeleton	LDB3	73.7	5			5			
F1NDN9	Keratin, type I cytoskeletal 19	cytoskeleton	KRT19	46.0	5	2	2		4	2	1
P13538	Myosin heavy chain, skeletal muscle, adult	cytoskeleton	not defined	223.0	6	36			33	21	
P02604	Myosin light chain 1, skeletal muscle isoform	cytoskeleton	not defined	20.9	6			5	1		
R9PXN5	Alpha-actinin-1 (Fragment)	cytoskeleton	ACTN1	101.5	6	15	8	13	8	4	1
P60706	Actin, cytoplasmic 1	cytoskeleton	ACTB	41.7	7	13	12	12	14	9	4
F1ND51	Uncharacterized protein	cytoskeleton	SPTBN1	272.2	7	8	1			1	1
F1P5V6	Uncharacterized protein	cytoskeleton	MYL3	21.9	8	7	7		2	1	1
P09207	Tubulin beta-6 chain	cytoskeleton	not defined	50.1	8	13	8		10	8	9
F1P493	Uncharacterized protein	cytoskeleton	MYH1A	223.2	9		48	151			
F1P3X4	Uncharacterized protein	cytoskeleton	MYH1F	222.9	9		41	135			
Q789A6	Nonmuscle myosin heavy chain	cytoskeleton	MYH10	228.9	9	13	18				2
E1BQZ4	Uncharacterized protein	cytoskeleton	EPB42	79.1	10		9		3	1	1
F1NHT3	Spectrin alpha chain, non-erythrocytic 1	cytoskeleton	SPTAN1	285.2	13	6	3		6		
F1P3X1	Uncharacterized protein	cytoskeleton	MYH1G	223.3	15	36	50	143	36	22	14

E1C2S1	Talin-1	cytoskeleton	TLN1	271.7	17	11	10		2	1	2
P16419	Myosin-binding protein C, fast-type	cytoskeleton	MYBPC2	126.9	19			21			
F1NNW0	Uncharacterized protein	cytoskeleton	MYOM2	163.3	19			19			
E1C3E2	Uncharacterized protein	cytoskeleton	MYOM1	182.0	19	7		15	1		
E1C5Z5	Uncharacterized protein	cytoskeleton	MYBPC1	129.9	20			21			
F1NSZ5	Uncharacterized protein	cytoskeleton	LAMA4	202.3	24	12	10		12	11	9
Q90688	Myosin-binding protein C, cardiac-type	cytoskeleton	MYBPC3	142.2	26	23	3	4	10	4	1
F1NDL1	Uncharacterized protein	cytoskeleton	NEB	306.6	28			30			
F1NCD4	Myosin-9	cytoskeleton	MYH9	226.5	38	22	41	10	16	3	
E1C523	Uncharacterized protein	cytoskeleton	MYH7B	198.2	50	27	19	78	23	21	6
F1NM49	Uncharacterized protein	cytoskeleton	MYH15	223.5	52	144	120	75	89	63	37
F1NGF6	Uncharacterized protein	cytoskeleton	not defined	419.9	60	6	3	58	4	2	3
P10587	Myosin-11	cytoskeleton	MYH11	228.7	72	53	68	12	27	33	10
F1P1D5	Uncharacterized protein	cytoskeleton	MYH7	221.7	77	47	25	56	82	71	26
R4GLP6	Uncharacterized protein (Fragment)	cytoskeleton	Gga.10533	3397.7	648	63	69	610	96	12	30
P15989	Collagen alpha-3(VI) chain	ECM	COL6A3	339.4	2	79	84	75	105	107	98
R4GIB6	Uncharacterized protein	ECM	MFAP2	20.6	2	1	2	1	1	1	
E1BT96	Uncharacterized protein	ECM	ASPN	42.1	2		2				
E1C203	Uncharacterized protein	ECM	DPT	23.5	2	2	2	1	2	2	
E1C7T4	Uncharacterized protein	ECM	PRELP	42.9	2		1	1			1
P12105	Collagen alpha-1(III) chain (Fragments)	ECM	COL3A1	121.2	2	1	1		2	1	1
F1P2F0	Collagen alpha-3(VI) chain	ECM	COL6A3	339.4	3		84	76	106	108	99
F1NJT4	Fibronectin	ECM	FN1	259.0	3		31	8	33	36	
Q01635	Laminin subunit beta-1 (Fragment)	ECM	LAMB1	34.8	3				4	4	3
R4GFV3	Uncharacterized protein	ECM	MFAP5	18.6	3		2		1	1	
F1NX60	Fibulin-1	ECM	FBLN1	78.1	3	1	1		2	2	1
F1NI79	Uncharacterized protein (Fragment)	ECM	COL5A1	180.1	3				2	3	3
E1C3A6	Uncharacterized protein	ECM	FBN2	313.2	3	4		1		1	
R4GM21	Uncharacterized protein	ECM	COL5A2	124.4	3	1	1		3	1	2
F1NJT3	Fibronectin	ECM	FN1	273.1	4	24	32		33	36	30

P17785	Annexin A2	ECM	ANXA2	38.6	5	3	1		2		
Q9W6H0	Mimecan	ECM	OGN	33.2	5	1	4	1	2	1	
F1NW38	Uncharacterized protein	ECM	COL18A1	137.3	5		1		1	3	3
F1P2R3	Uncharacterized protein	ECM	COL4A1	160.6	7	7	6	4	4	6	5
E1BRE9	Decorin	ECM	DCN	39.6	9	2	9	4	5	6	2
F1P2Q3	Uncharacterized protein	ECM	COL4A2	168.8	12	10	9	5	12	11	8
F1NJ23	Laminin subunit beta-1	ECM	LAMB1	168.4	13	8	1	1	3	3	2
P02457	Collagen alpha-1(I) chain	ECM	COL1A1	137.7	13	7	6	2	8	10	6
F1NI05	Uncharacterized protein	ECM	LAMC1	119.3	14	9	7	3	8	7	7
F1P0H9	Collagen alpha-2(I) chain	ECM	COL1A2	128.8	16	14	8	8	11	11	12
F1NP23	Uncharacterized protein	ECM	NID1	136.5	23	16	15	4	8	8	7
E1C700	Uncharacterized protein	ECM	LAMB2	179.0	27	17	19	5	19	20	10
R4GKA6	Collagen alpha-2(VI) chain	ECM	COL6A2	102.4	36	25	25	18	27	31	26
F1NX22	Collagen alpha-1(XII) chain	ECM	COL12A1	339.6	36	3	10		25	20	17
F1NFE0	Collagen alpha-1(VI) chain	ECM	COL6A1	107.9	41	26	24	16	26	30	29
F1NII7	Uncharacterized protein	ECM	FBN1	301.1	45	39	16	21	6	17	7
F1NVM0	Uncharacterized protein	membrane	B-G	21.1	2	1		1	1	2	
F1NFG6	Uncharacterized protein	membrane	RHAG	43.8	2	1	1		2	2	1
R4GHN3	Uncharacterized protein	membrane	PLVAP	47.0	2	2					
F1NHT7	Uncharacterized protein	membrane	CTNNB1	85.4	2	2					
E1C6L8	Uncharacterized protein	membrane	MCAM	64.2	2	1	1				
F1NYZ4	Uncharacterized protein	membrane	SLC8A1	105.8	2	2					
R4GHJ7	Uncharacterized protein	membrane	PHKB	123.0	2			2			
F1NRB7	Sodium/potassium-transporting ATPase subunit beta	membrane	ATP1B1	28.5	3	3	1		1		
P84173	Prohibitin	membrane	PHB	29.9	3	1	3		1		
P63247	Guanine nucleotide-binding protein subunit beta-2-like 1	membrane	RACK1	35.1	3	3	1				
A0M8T8	Caveolin	membrane	CAV1	20.5	4	2	3		1	2	
F1N833	Prohibitin-2 (Fragment)	membrane	PHB2	30.6	4	2	1	2	2	1	
P17790	Basigin	membrane	BSG	42.4	4	3		1	1		
F1NER9	Uncharacterized protein	membrane	CD36	52.6	4	3	3			2	
F1NQ78	Uncharacterized protein	membrane	MYLK	210.3	4	4					
F1N8N7	Integrin beta	membrane	ITGB1	88.6	5	5					

H9KZZ3	Uncharacterized protein	membrane	not defined	111.9	5	2	5		3	2	2
E1BTV1	Uncharacterized protein (Fragment)	membrane	STOM	31.4	6		4		5	1	1
F1NSY1	Sodium/potassium-transporting ATPase subunit alpha (Fragment)	membrane	ATP1A1	112.0	7	7					
F1NDL4	Uncharacterized protein	membrane	not defined	130.9	9	4	7		1		2
P15575	Band 3 anion transport protein	membrane	SLC4A1	102.2	14	12	9	4	10	8	6
F1NSS0	Uncharacterized protein	membrane	LOC101749159	265.4	19	8	12	3	5	5	3
P84229	Histone H3.2	nuclear	H3-I	15.4	2	1			2		
F1NSP8	Uncharacterized protein	nuclear	HNRNPU	74.9	2	2					
P02263	Histone H2A-IV	nuclear	not defined	13.9	3		2	1	3	1	1
F1NT78	Uncharacterized protein	nuclear	not defined	22.0	3		3				
R4GKX4	Uncharacterized protein (Fragment)	nuclear	HNRNPA3	25.5	3	1	3				
Q5ZME1	Uncharacterized protein	nuclear	HNRNPA2B1	37.0	3	3	3		1		
F1NEG6	Uncharacterized protein	nuclear	HNRNPH1	56.5	3	1	3	1			
Q5ZHZ0	Spliceosome RNA helicase DDX39B	nuclear	DDX39B	49.0	3	1	2	1		1	
E1C2R8	Uncharacterized protein (Fragment)	nuclear	DDX17	75.1	3	3	2		1	1	
F1P555	Uncharacterized protein	nuclear	SFPQ	69.5	3	3	3				
P13648	Lamin-A	nuclear	LMNA	73.1	3				3		
P02259	Histone H5	nuclear	not defined	20.7	4	3	2		1	2	2
F1N9S7	Annexin	nuclear	ANXA1	38.5	4			1	3		
E1BZE6	Uncharacterized protein	nuclear	LOC100859627	40.8	4	3	4		3	1	
F1N9H4	Elongation factor 1-alpha	nuclear	EEF1A2	50.5	5	6		7	6		3
P0C1H3	Histone H2B 1/2/3/4/6	nuclear	H2B-I	13.9	6	5	4	2	6	4	5
P62801	Histone H4	nuclear	H4-I	11.4	8	6	7	3	5	5	6
O73790	Heterochromatin-associated protein MENT	nuclear	SERPINB10	47.4	9				9		
F1NIX2	Uncharacterized protein	nuclear	DDX3X	72.6	9	8	3	2			
P55807	NAD(P)(+)--arginine ADP-ribosyltransferase 2	secreted	not defined	34.9	2	1			2	1	
F1NDN6	Uncharacterized protein	secreted	KRT12	54.3	2	3	2	1	3	3	

E1C7P4	Uncharacterized protein	secreted	CFH	147.8	2	1	1				
P20763	Ig lambda chain C region	secreted	not defined	11.4	3		1		3	2	1
P30374	Ribonuclease homolog	secreted	not defined	15.9	3				3		
F1NPJ8	Glutathione peroxidase (Fragment)	secreted	GPX3	24.6	3	1	2		1	3	2
F1NYI2	Uncharacterized protein	secreted	FBP2	37.1	3			3			
P19121	Serum albumin	secreted	ALB	69.9	3				1	1	1
E1C677	Uncharacterized protein	secreted	Gga.18306	17.3	4	2	1	1	3	1	
E1C7A7	Uncharacterized protein	secreted	VTN	51.6	4		3		3		
F1P587	Uncharacterized protein	secreted	LOC418892	189.9	4	2				1	1
P01875	Ig mu chain C region	secreted	not defined	48.1	5		2		3		2
P08250	Apolipoprotein A-I	secreted	APOA1	30.7	6	2	2	1	5	4	3
E1C1G8	Uncharacterized protein	secreted	FBLN5	49.6	6	4	5	2	6	5	3
R4GJS6	Uncharacterized protein	secreted	C3	151.1	6	1			3	2	1
F1NK40	Uncharacterized protein	secreted	A2ML4	163.2	6	3	2	1	3	3	3
P02001	Hemoglobin subunit alpha-D	secreted	HBAD	15.7	7	5	5	3	7	5	3
R4GJ67	Uncharacterized protein	secreted	not defined	29.0	7	4	4	1	4	2	5
E1C6M8	Uncharacterized protein	secreted	EFEMP1	50.6	7	3	4	1	2	2	
P01994	Hemoglobin subunit alpha-A	secreted	HBAA	15.4	8	7	5	4	8	6	6
F1NWX6	Plasminogen	secreted	PLG	90.7	8	4	7			1	1
E1C6J4	Uncharacterized protein	secreted	VWF	309.5	8	4	4		4	1	1
F1N8G6	Uncharacterized protein	secreted	TINAGL1	51.6	10	6	10		1	1	
F1NEF7	Uncharacterized protein	secreted	LECT2	35.2	12	10	5		5		
F1P4V1	Fibrinogen alpha chain	secreted	FGA	82.3	20	12	12	7	12	11	12
F1P494	Uncharacterized protein (Fragment)	secreted	TGFBI	70.4	22	5	6	4	12	18	13
E1BV78	Uncharacterized protein	secreted	FGG	49.9	25	20	23	10	16	17	11
F1NZZ2	Uncharacterized protein	secreted	not defined	379.6	26	10	10	3	13	15	6
Q02020	Fibrinogen beta chain (Fragment)	secreted	FGB	52.6	32	27	27	10	18	19	16
Q5ZIH4	Uncharacterized protein	uncharacterized	Gga.1330	16.5	2	2	1		1		
R4GLW5	Uncharacterized protein	uncharacterized	LOC426257	34.2	2	1	2		1	1	
E1BYE0	Sulfotransferase (Fragment)	uncharacterized	LOC415852	34.4	2	2					
F1P0X3	Uncharacterized protein	uncharacterized	SEPT11	50.6	2	2	1				1
F1P0Y7	Uncharacterized protein (Fragment)	uncharacterized	not defined	73.2	2		2				

R4GL00	Uncharacterized protein	uncharacterized	LOC425663	20.1	3	2			2	1	
P28318	Protein MRP-126	uncharacterized	not defined	14.1	4	4	4	1	4	2	1
E1BV34	Uncharacterized protein	uncharacterized	Gga.3145	68.5	4	3	1		2		1
R4GMA5	Uncharacterized protein	uncharacterized	not defined	26.7	14	12	10	3	9	9	6
F6U7Z0	Uncharacterized protein	uncharacterized	not defined	308.9	42			44			
