# Supplementary material for: Avian lungs: A novel scaffold for lung bioengineering
Source: PLoS One. 2018 Jun 27;13(6):e0198956. doi: 10.1371/journal.pone.0198956 (PMC6021073; doi:10.1371/journal.pone.0198956)
Supplement: S2 Table — (PDF) [file pone.0198956.s007.pdf]

S2 Table: Total peptide counts for positively identified proteins in individual emu lung samples.

Accession Number	Protein description	Subcellular location	Gene symbol	Molecular weight [kDa]	Σ# Unique Peptides	Emu1	Emu2	Emu3	Emu4
C6L8R0	Chain C, Crystal Structure Of Emu Hemoglobin At 2.3 Angstrom Resolution	cytoplasm	HBAA-1	15.5	9	5	7	6	9
A0A075B5G0	Chain D, Crystal Structure Of Emu Hemoglobin At 2.3 Angstrom Resolution	cytoplasm	HBAA-2	16.3	13	5	12	5	13
P86874	Myoglobin	cytoplasm	MB	17.5	5			3	5
I7E848	titin, partial	cytoplasm	TTN	17.5	2				2
Q9B6V7	NADH dehydrogenase subunit 1 (mitochondrion)	cytoplasm	NADH dehydrogenase subunit 1	35.9	2			1	2
O03891	cytochrome oxidase II, partial (mitochondrion)	cytoplasm	COX2	22.3	4				4
Q9B6U8	NADH dehydrogenase subunit 5 (mitochondrion)	cytoplasm	NADH dehydrogenase subunit 5	66.5	2				2
Q9B6U9	NADH dehydrogenase subunit 4 (mitochondrion)	cytoplasm	NADH dehydrogenase subunit 4	51.0	2				2
G8HY07	beta actin	cytoskeleton	ACTB	41.7	21	8	21	14	17
B4ZAC9	eukaryotic translation elongation factor 2, partial	nuclear	EEF2	21.0	2		2		1
B4ZCL0	fibrinogen beta chain, partial	secreted	FGB	11.5	7	1	2	4	6
B4ZBR4	fibrinogen beta chain, partial	secreted	FGB	2.9	2	1	1	1	2
H9NA73	adiponectin	secreted	ADIPOQ	26.5	2	1		1	2
E2RUJ8	ovotransferrin precursor	secreted	ovotransferrin precursor	77.8	2	2			
